# Supplementary figures and images for: Inhibition of ribosome biogenesis in the epidermis is sufficient to trigger organism-wide growth quiescence independently of nutritional status in C. elegans
Source: PLoS Biol. 2023 Aug 31;21(8):e3002276. doi: 10.1371/journal.pbio.3002276 (PMC10499265; doi:10.1371/journal.pbio.3002276)

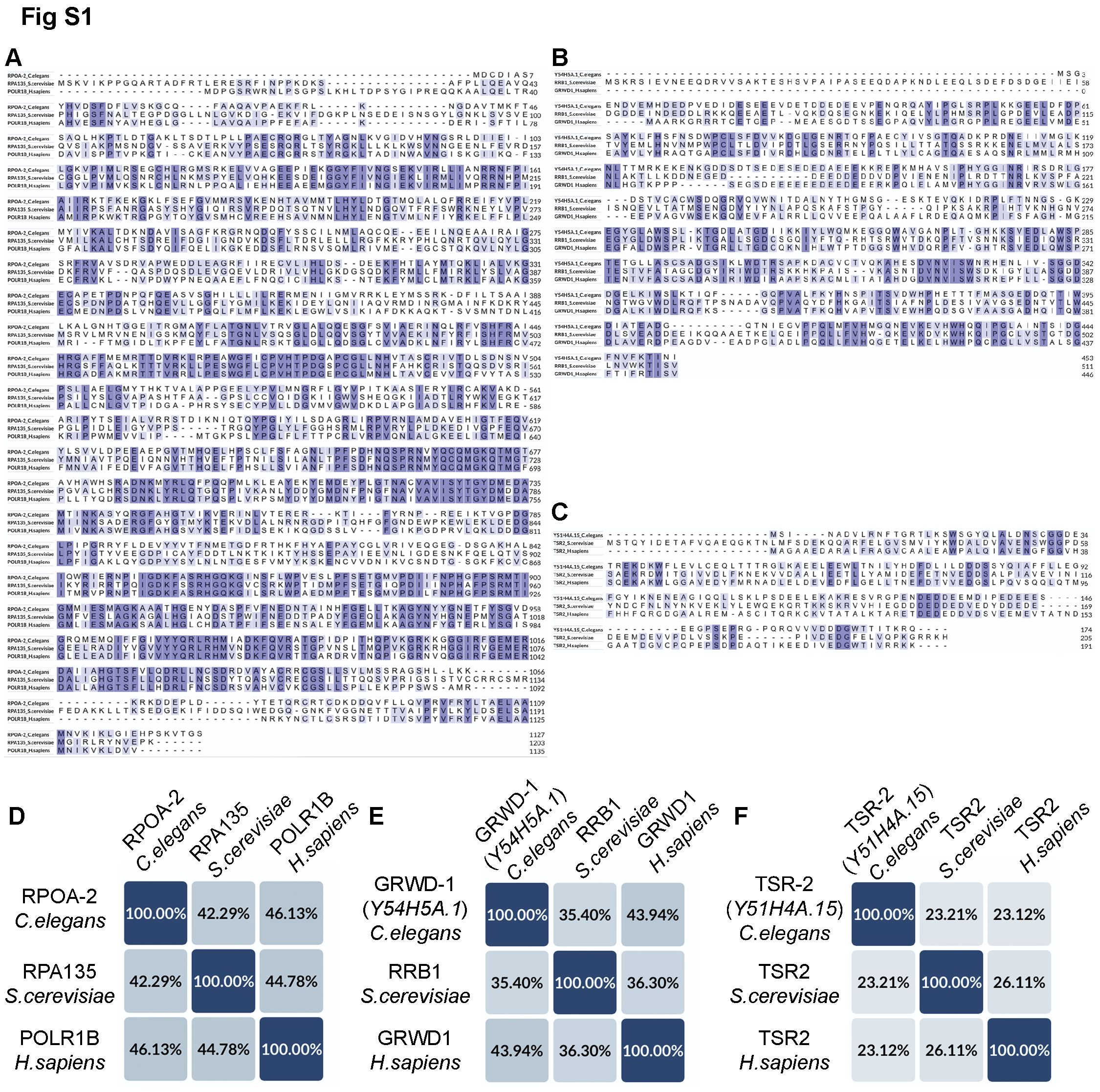

Supplement: S1 Fig — (A-C) Amino acid sequence alignments of ribosome biogenesis factors from 3 different species, Caenorhabditis elegans, Saccharomyces cerevisiae, and Homo sapiens. Alignments were performed using UniProt align function. (A) RPOA-2 in C. elegans shows homology to yeast RPA135 and human POLR1B. (B) GRWD-1 encoded by Y54H5A.1 in C. elegans is homologous to yeast RRB1 and human GRWD1. (C) TSR-2 encoded by Y51H4A.15 in C. elegans is homologous to yeast Tsr2 and human TSR2. (D-F) Comparison of the identity of RPOA-2 (D), GRWD-1 (E), and TSR-2 (F) in C. elegans with their homologues from S. cerevisiae and H. sapiens. (TIF) [file pbio.3002276.s001.tif]

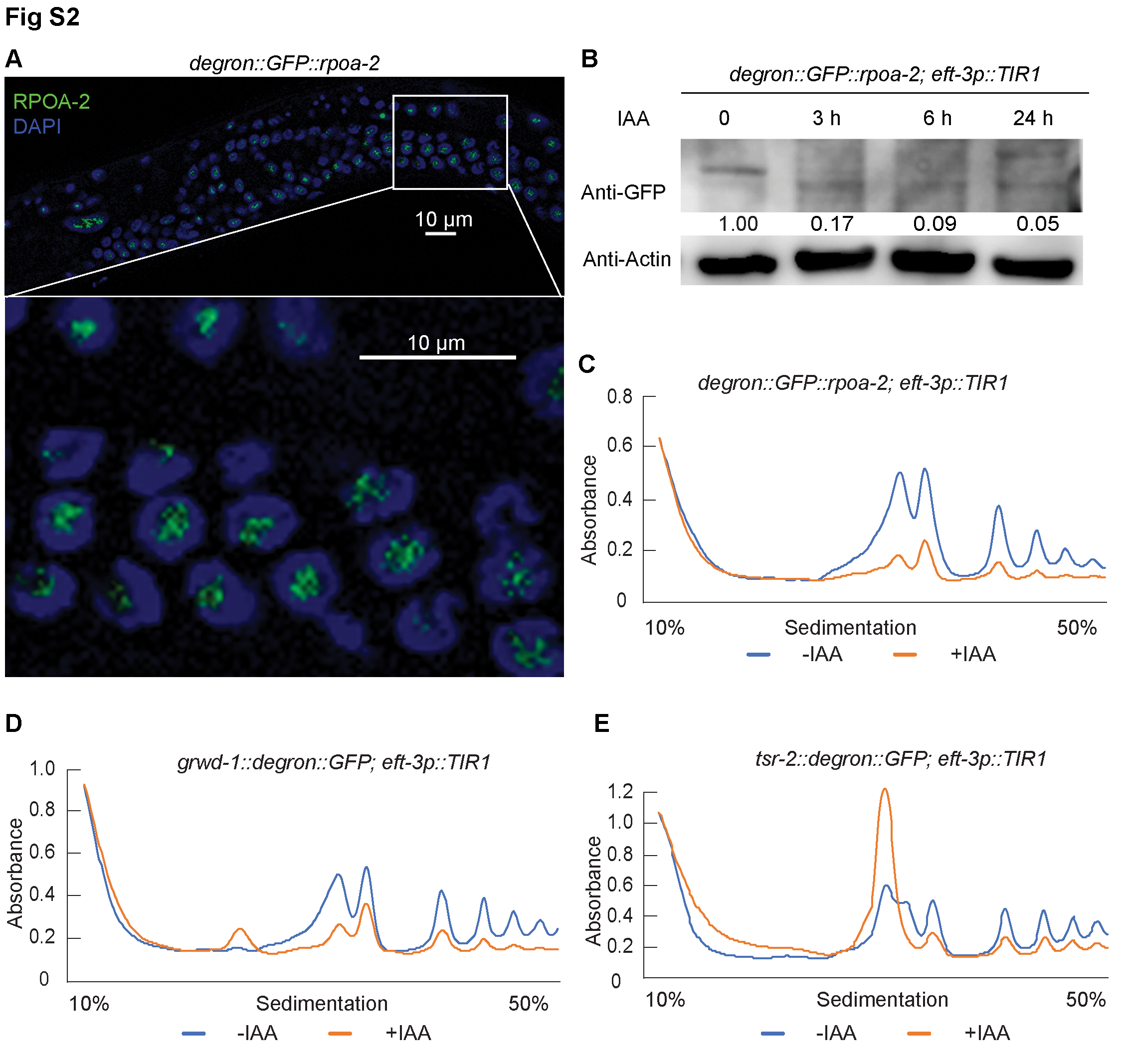

Supplement: S2 Fig — (A) DAPI staining of L4 stage animals expressing degron::GFP-integrated RPOA-2. RPOA-2 is enriched in the nucleoli. (B) L4 stage animals expressing degron::GFP-integrated RPOA-2 and TIR1 ubiquitously were treated with 1 mM IAA. Animals were collected and lysed at 4 time points, 0-hour (0), 3-hour (3 h), 6-hour (6 h), and 24-hour (24 h). Western blots were performed using antibodies against GFP and Actin. The relative RPOA-2 protein levels were quantified using Fiji software. The numbers above the gel lanes represent the relative protein level normalized to Actin. (C-E) RPOA-2, GRWD-1, and TSR-2 are necessary for ribosome biogenesis. Polysome profiles of degron::GFP::rpoa-2; eft-3p::TIR1 (C), grwd-1::degron::GFP; eft-3p::TIR1 (D), and tsr-2::degron::GFP; eft-3p::TIR1 (E) strains treated with and without 1 mM IAA for 24 hours from the L4 stage. The depletion of RPOA-2, GRWD-1, or TSR-2 by the AID system caused a dramatic decrease of ribosomes and polysomes. The underlying data for (C-E) can be found in Tab I in S1 Data. (TIF) [file pbio.3002276.s002.tif]

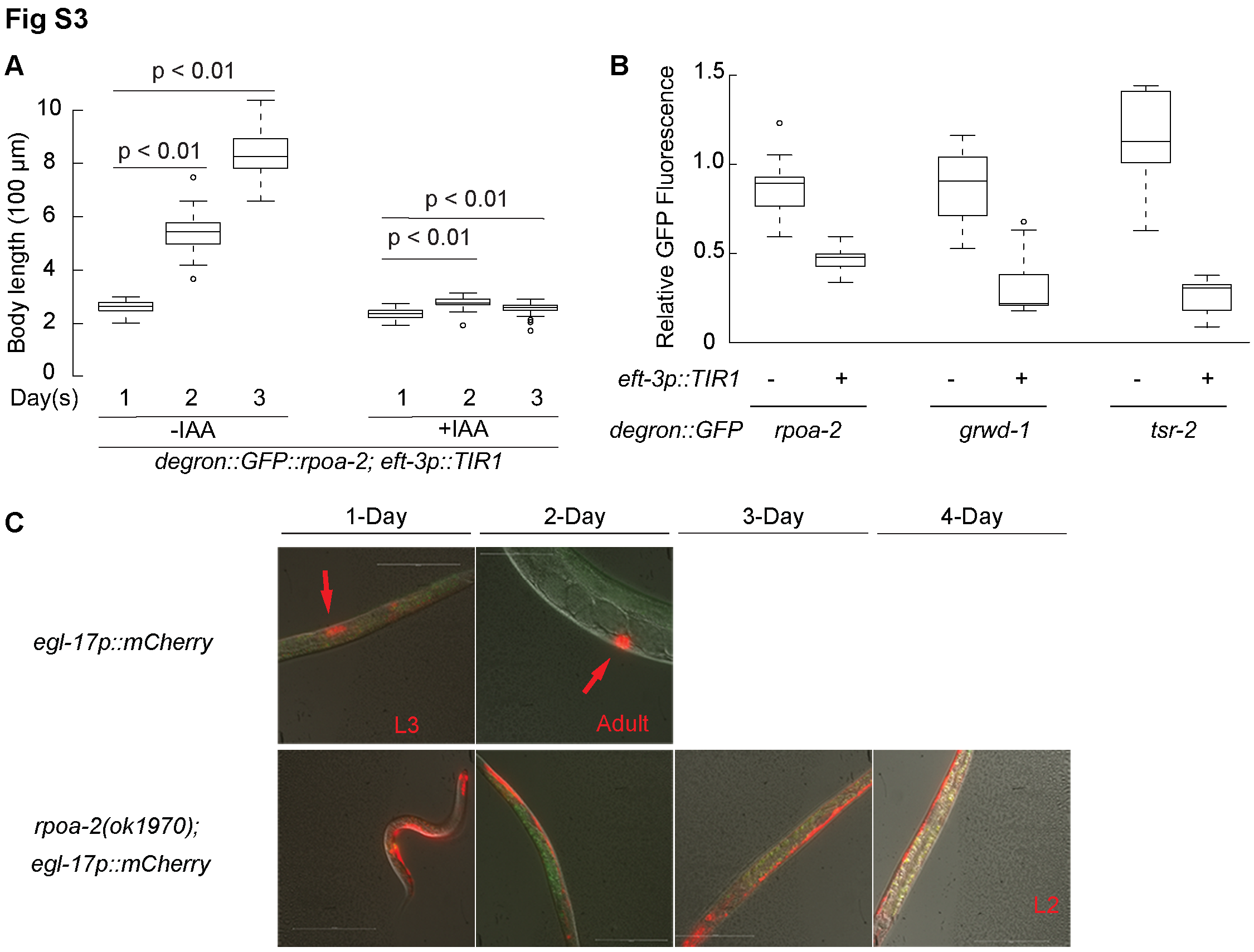

Supplement: S3 Fig — (A) Embryos of the degron::GFP::rpoa-2; eft-3p::TIR1 strain were treated with and without 1 mM IAA, and body length was measured over a span of 3 days with 40 animals for each condition per day. P values were calculated using an independent t test and adjusted by Bonferroni correction. (B) Basal degradation of RPOA-2, GRWD-1, and TSR-2 were detected by GFP fluorescence intensity in strains expressing global TIR1 compared to those without TIR1 expression. Higher degradation of TSR-2 with TIR1 was observed compared to that of RPOA-2 or GRWD-1. GFP fluorescence was measured from 12 L4 stage animals of degron::GFP-integrated RPOA-2, GRWD-1, or TSR-2 strains. (C) Vulva invariant cell lineage was not observed in rpoa-2(ok1970) animals after 4 days from the L1 stage. Arrows indicate vulva invariant cell lineage in wild type. The underlying data for (A, B) can be found in Tab J in S1 Data. (TIF) [file pbio.3002276.s003.tif]

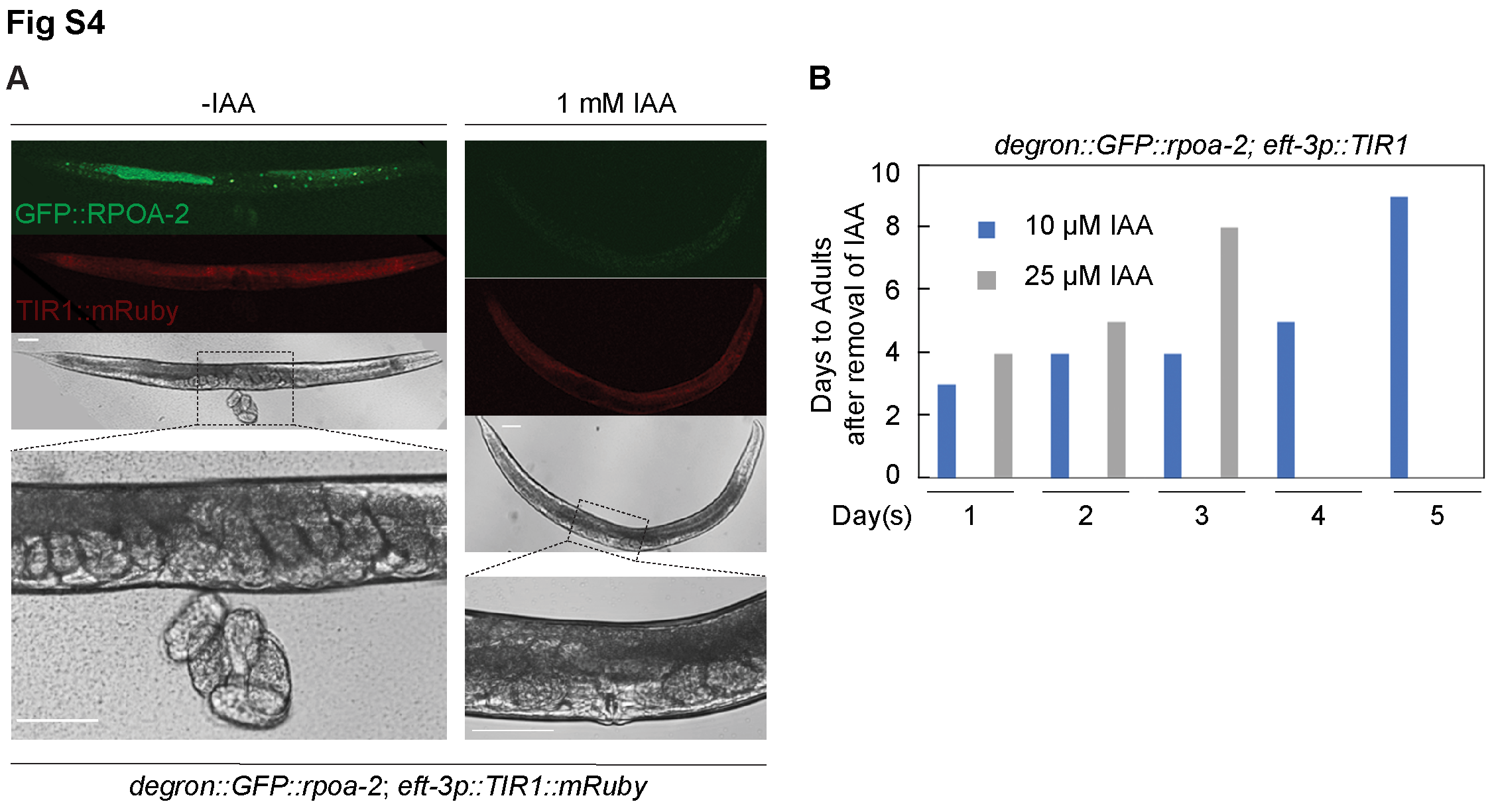

Supplement: S4 Fig — (A) L4 stage animals were treated with (1 mM) and without (−) IAA for 24 hours. Animals were immobilized on slides using 1 mM levamisole. All these animals grew to gravid adults. Scale bar, 50 μm. (B) Growth reversibility was tested by treating embryos of degron::GFP::rpoa-2; eft-3p::TIR1 with 10 μM and 25 μm IAA from 1 to 5 days (x-axis) and then transferring them to plates without IAA. The presence of gravid adults and F1 progeny on plates was inspected daily and the number of days taken to reach fertile adulthood was recorded (y-axis). No bar indicates that no gravid adults or F1 progeny were observed after removal of IAA. The underlying data for (B) can be found in Tab K in S1 Data. (TIF) [file pbio.3002276.s004.tif]

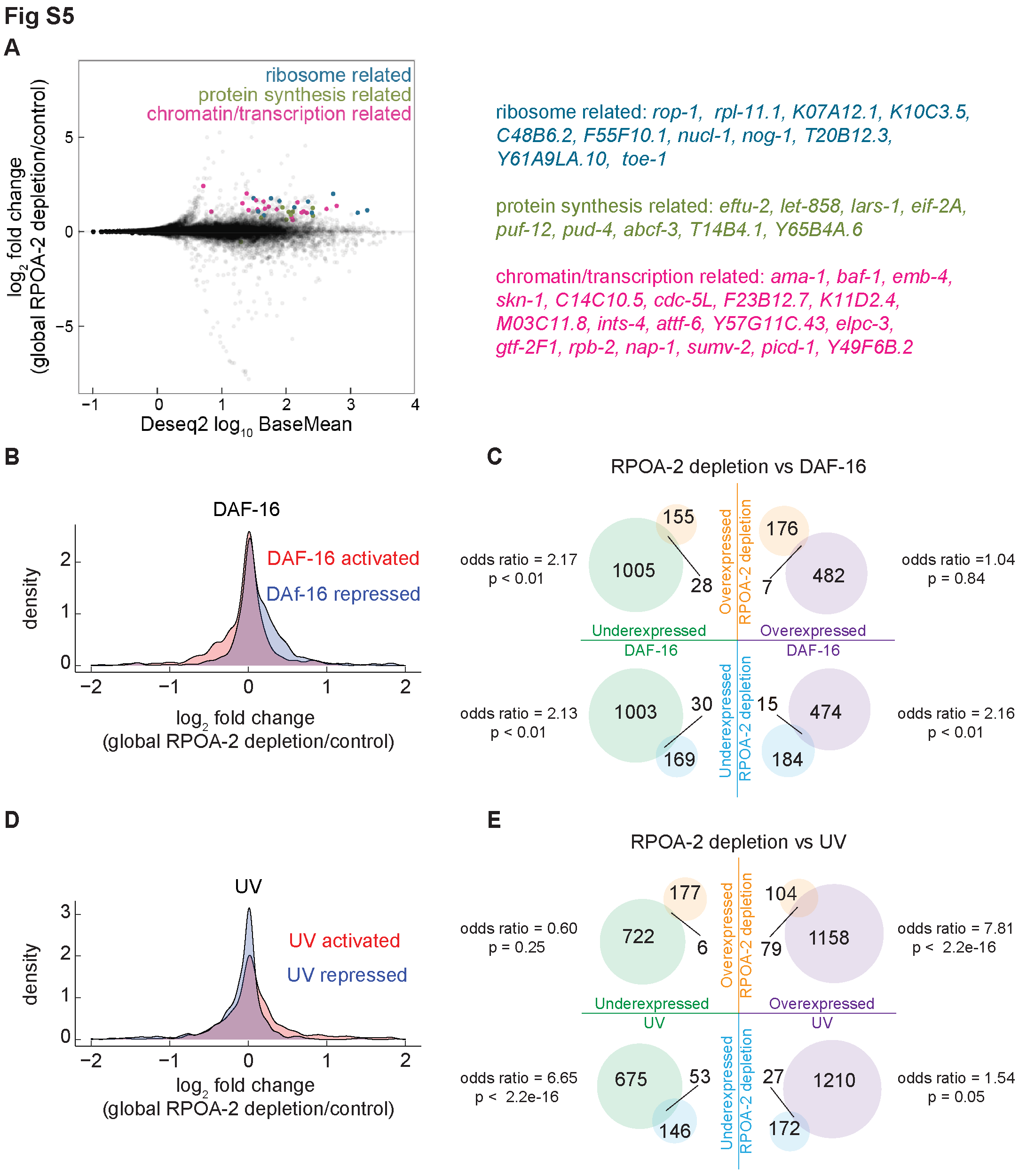

Supplement: S5 Fig — (A) Three representative significant GO categories with respective genes log2 fold changes were plotted. The light blue, green, and magenta points indicate ribosome, protein synthesis, and chromatin/transcription-related genes, respectively. (B) Deseq2 log2 fold change values in response to global RPOA-2 depletion were plotted for overexpressed (light red) and underexpressed (light blue) DAF-16 target genes [50]. (C) Shared gene expression changes in response to RPOA-2 depletion by RNA-seq and DAF-16 target genes were shown in the Venn diagrams. (D) Deseq2 log2 fold change values in response to global RPOA-2 depletion were plotted for overexpressed (light red) and underexpressed (light blue) UV response genes [51]. (E) Shared gene expression changes in response to RPOA-2 depletion by RNA-seq and UV response genes were shown in the Venn diagrams. The underlying data for (B-E) can be found in Tab L in S1 Data. (TIF) [file pbio.3002276.s005.tif]

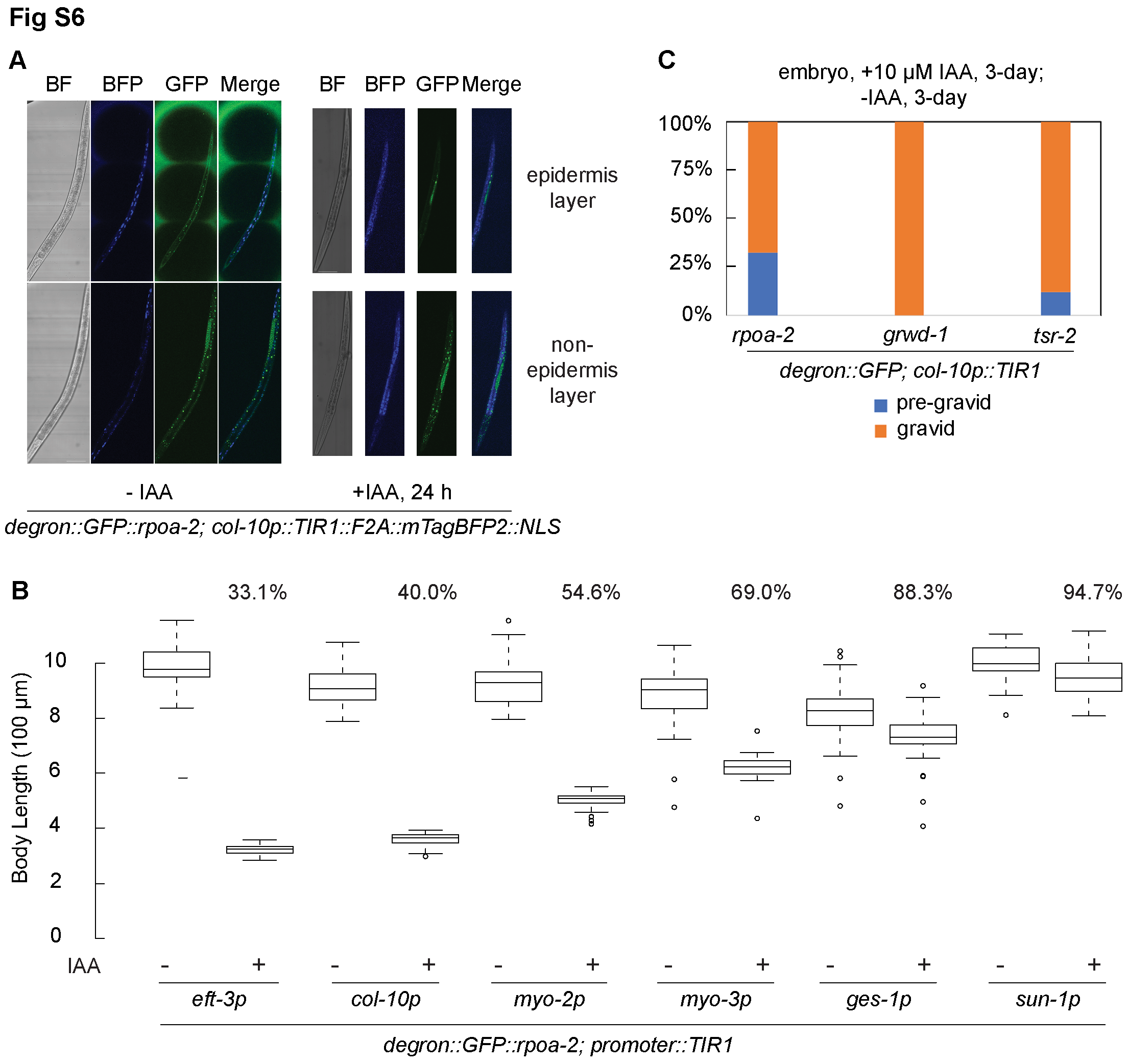

Supplement: S6 Fig — (A) GFP displayed the expression pattern of RPOA-2 and BFP showed the TIR1 expression in epidermis driven by col-10 promoter. After 1 mM IAA treatment for 24 hours, RPOA-2 was specifically depleted in the epidermis. (B) Synchronized embryos of strains expressing degron::GFP-integrated RPOA-2 and TIR1 in specific tissues were treated with and without 1 mM IAA for 3 days. Body length was measured using Fiji software. Data were obtained from 34 animals without IAA and 50 animals with IAA treatment for each strain. (C) Embryos expressing a degron::GFP-integrated ribosome biogenesis factor (RPOA-2, GRWD-1, or TSR-2) and TIR1 in epidermis (col-10p) were exposed to 10 μM IAA for 3 days and then transferred on plates without IAA for another 3 days. The percentage of animals that recovered back to gravid adults were measured (n = 40). Animals were immobilized by 1 mM levamisole. The underlying data for (B, C) can be found in Tab M in S1 Data. (TIF) [file pbio.3002276.s006.tif]

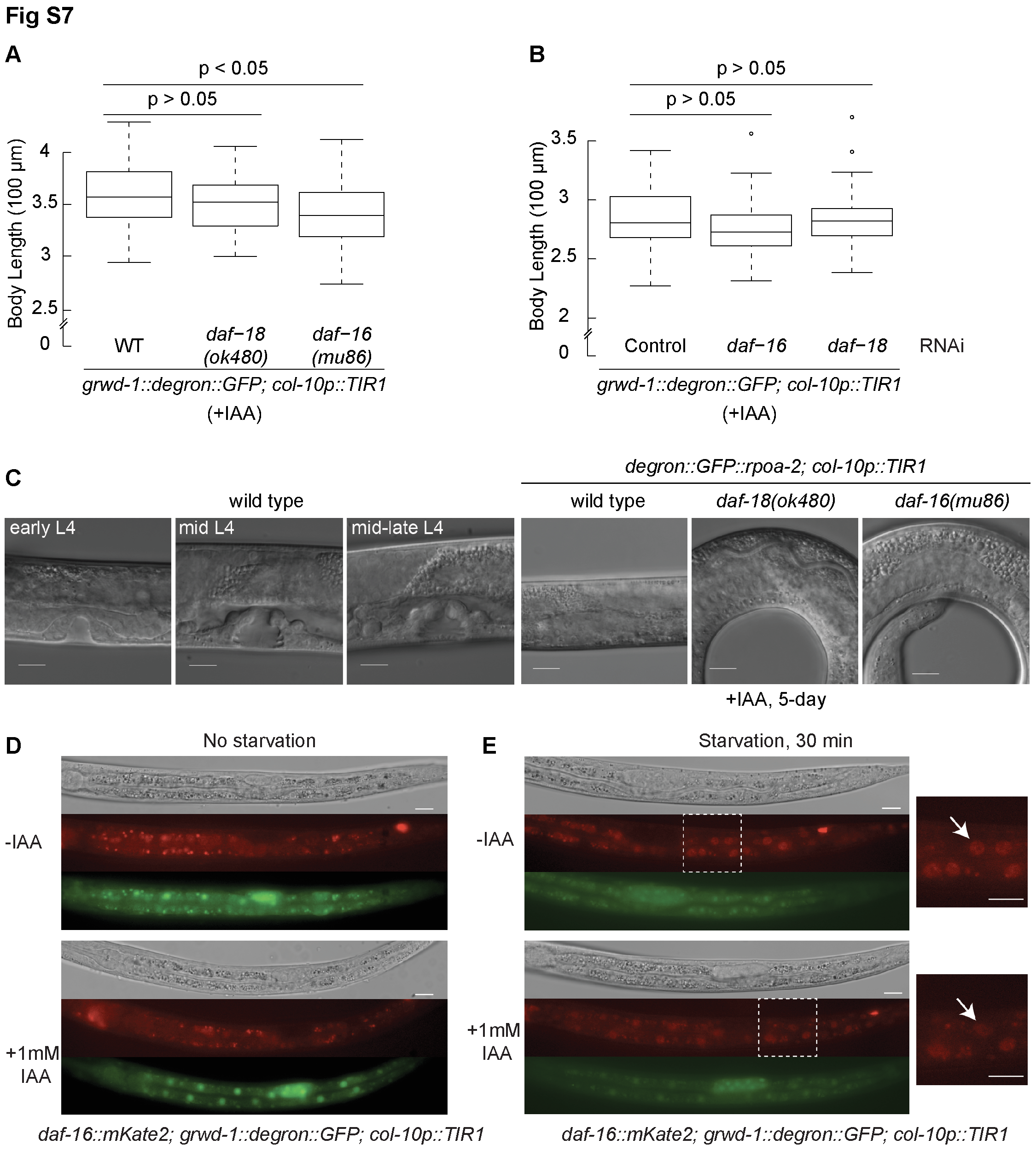

Supplement: S7 Fig — (A) Animals of daf-18(ok480) and daf-16(mu86) did not show larger growth compared to wild type when the epidermal ribosome biogenesis was inhibited (grwd-1::degron::GFP; col-10p::TIR1, +IAA). Data are expressed as body length measured from 3 independent experiments with at least 18 animals in each replicate. (B) daf-16 or daf-18 RNAi did not affect animal growth in the absence of new epidermal ribosomes. Synchronized embryos were grown on NGM with 1 mM IAA for 3 days. Data are expressed as body length measured from 40 worms. P values were calculated using an independent t test and adjusted by Bonferroni correction in (A, B). (C) The vulval extracellular space (indicative of transition into L4 stage) was not observed in daf-18(ok480) and daf-16(mu86) mutants when epidermal ribosome biogenesis was inhibited from embryos for 5 days. (D) Representative images of strain daf-16::mKate2; grwd-1::degron::GFP; col-10p::TIR1 that were grown from embryos on NGM with and without 1 mM IAA for 24 hours. (E) When these animals (in D) were transferred to survival NGM without E. coli for 30 minutes, animals in both conditions showed nuclear localization of DAF-16::mKate2. Animals were immobilized on slides using 20 mM sodium azide. Arrows indicate nuclear DAF-16 localization. Scale bar, 10 μm. The underlying data for (A, B) can be found in Tab N in S1 Data. (TIF) [file pbio.3002276.s007.tif]

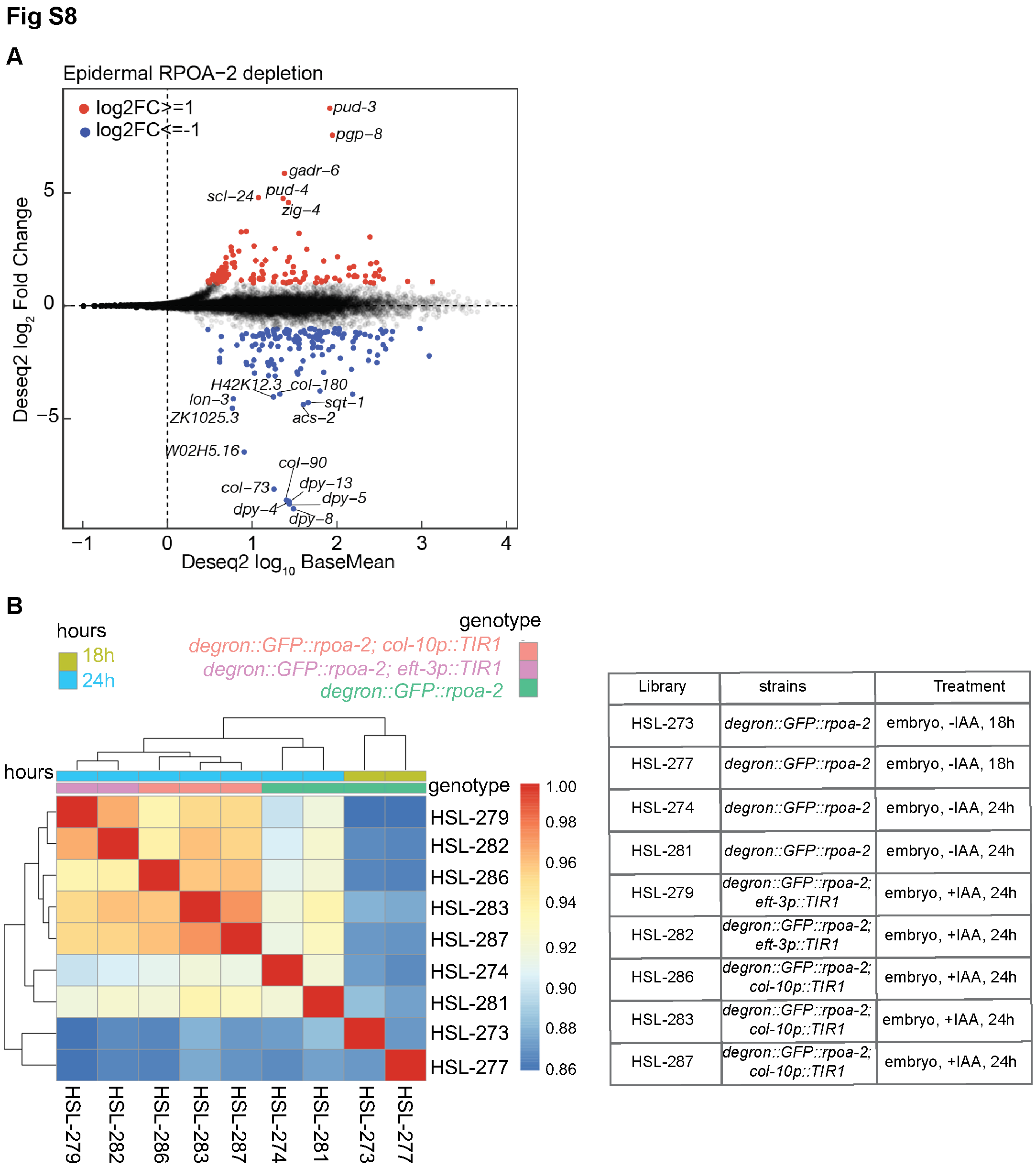

Supplement: S8 Fig — (A) Log2 fold changes of coding genes (y-axis) in response to epidermal RPOA-2 depletion (degron::GFP::rpoa-2; col-10p::TIR1) were plotted with respect to control (degron::GFP::rpoa-2) (x-axis). Log2 fold changes and base mean values were calculated using Deseq2. Genes showing more than 2-fold differential expression were marked in red (overexpressed) and blue (underexpressed), and symbols indicate genes that were differentially expressed at least 16-fold. (B) Spearman correlation across different replicates was plotted using a clustered heatmap. The underlying data can be found in the S1 Table. (TIF) [file pbio.3002276.s008.tif]

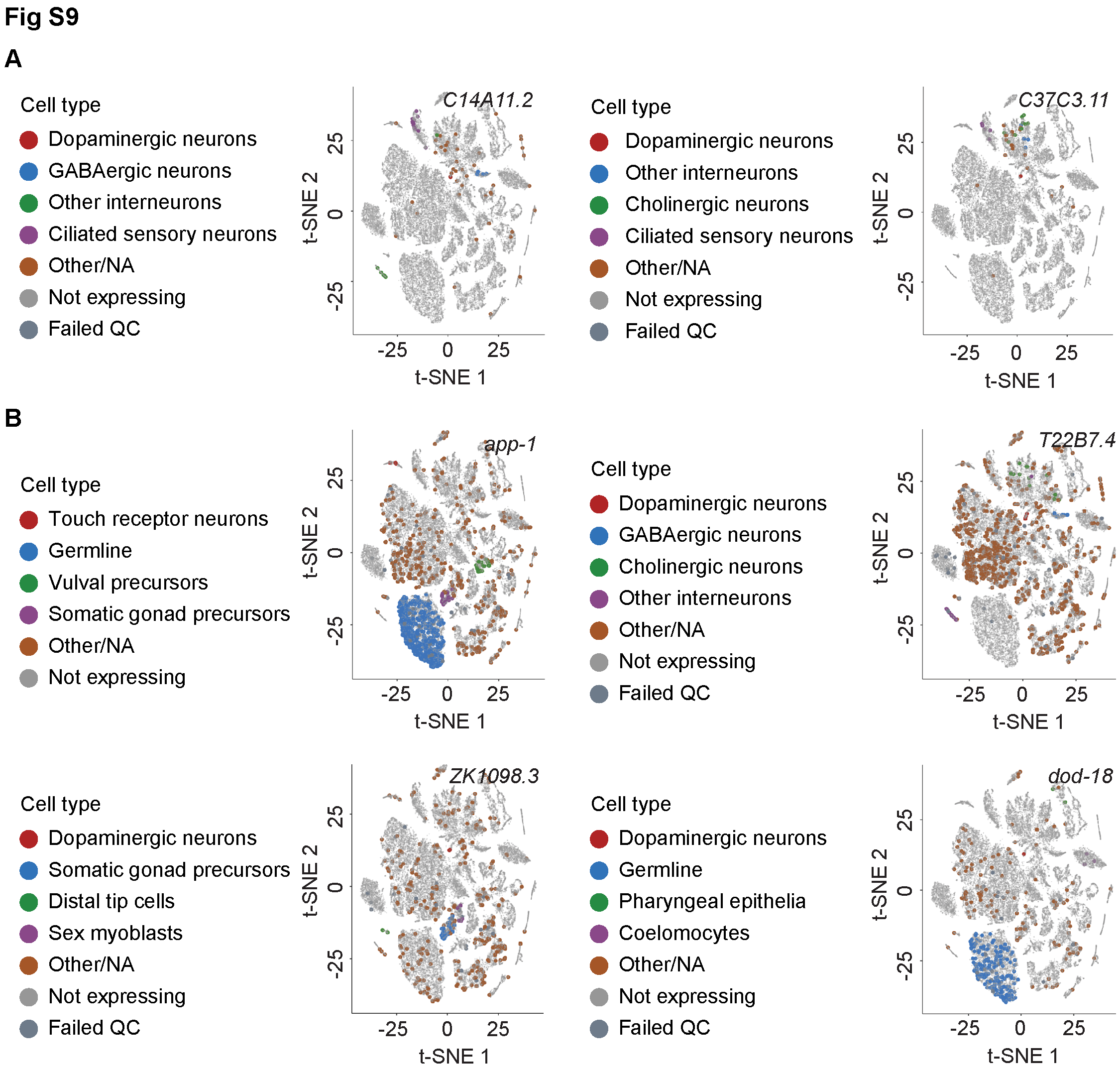

Supplement: S9 Fig — Single-cell t-SNE plots for (A) 2 selected underexpressed genes (C14A11.2 and C37C3.11) and (B) 4 selected overexpressed genes (app-1, T22B7.4, ZK1098.3, and dod-18). The t-SNE plots were generated using L2 stage single-cell RNA-seq data and single-cell-worm RNA software [57]. The colored points from t-SNE plots were original; however, their size was enlarged to ease visualization. The underlying data can be found in Tab O in S1 Data. (TIF) [file pbio.3002276.s009.tif]

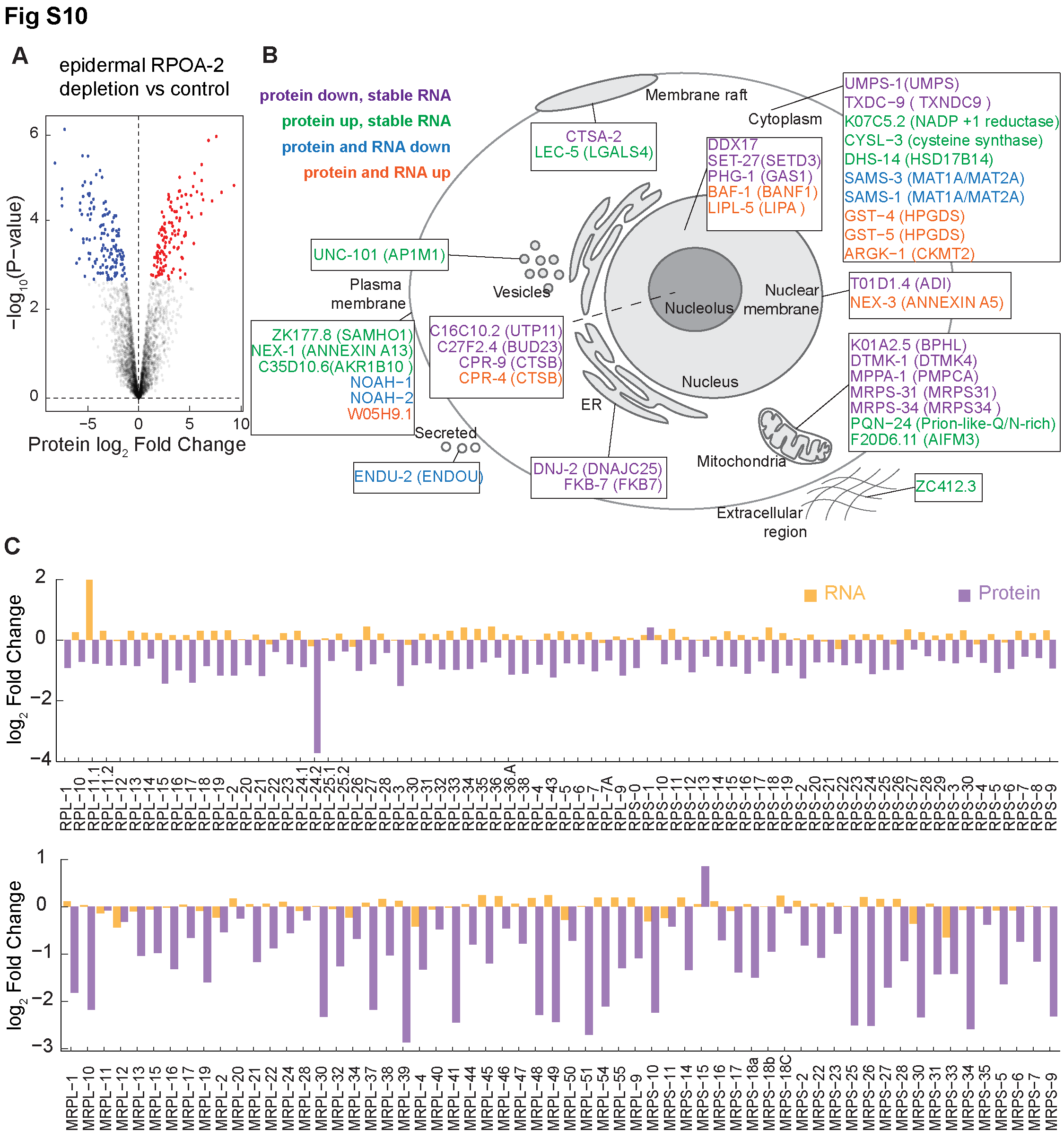

Supplement: S10 Fig — (A) Label-free intensity (LFQ) based mass spectrometry quantification of proteins in response to the epidermal RPOA-2 depletion using the DEP package. Proteins showing more than 2-fold over- and underexpression were marked in red and blue, respectively. (B) Summary of the cellular location and function of differentially expressed proteins in response to RPOA-2 depletion in the epidermis. (C) Bar charts showing the expression of cytoplasmic and mitochondrial ribosomal protein genes at the RNA (orange) and protein (purple) levels. The underlying data for (C) can be found in Tab P in S1 Data. (TIF) [file pbio.3002276.s010.tif]

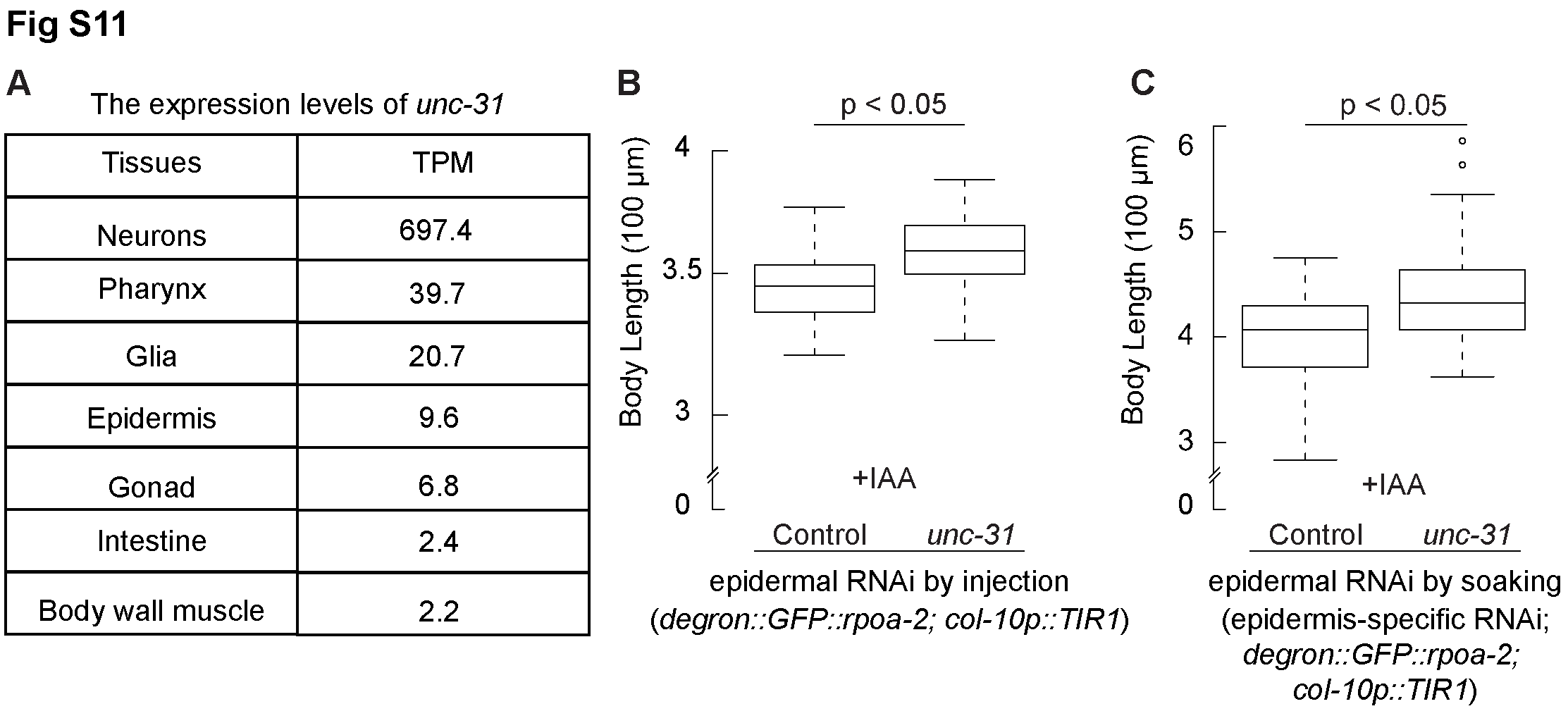

Supplement: S11 Fig — (A) Respective TPM (transcripts per million) values of unc-31 gene in different tissues were plotted using single-cell expression data from L2 animals [57]. (B) Sense and antisense DNA fragments targeting unc-31 gene and control gene (mKate2) driven by wrt-2 promoter were injected to the inducible epidermal ribosome biogenesis inhibition strain. Animals with reduced UNC-31 grew significantly larger compared to control when the epidermal ribosome biogenesis was inhibited (degron::GFP::rpoa-2; col-10p::TIR1, +IAA) from embryos for 3 days. Data were obtained from 3 independent experiments with 16 animals for each replicate. (C) Double-strand RNA targeting the unc-31 gene and control gene (wrmScarlet) transcribed in vitro was used to soak L1 larvae of epidermis-specific RNAi and epidermis-specific inducible ribosome biogenesis strain for 24 hours. The soaked larvae were then transferred to NGM plates with IAA for 3 days. Reducing unc-31 expression by soaking significantly increased worm body length compared to the control. Animals were immobilized using 0.5% 1-phenoxy-2-propanol. Each 5× image was analyzed by custom MATLAB script (S1 Text). Data were obtained from 3 independent experiments with 27 animals for each replicate. Statistical significance was determined using an independent t test. The underlying data for (B, C) can be found in Tab R in S1 Data. (TIF) [file pbio.3002276.s011.tif]

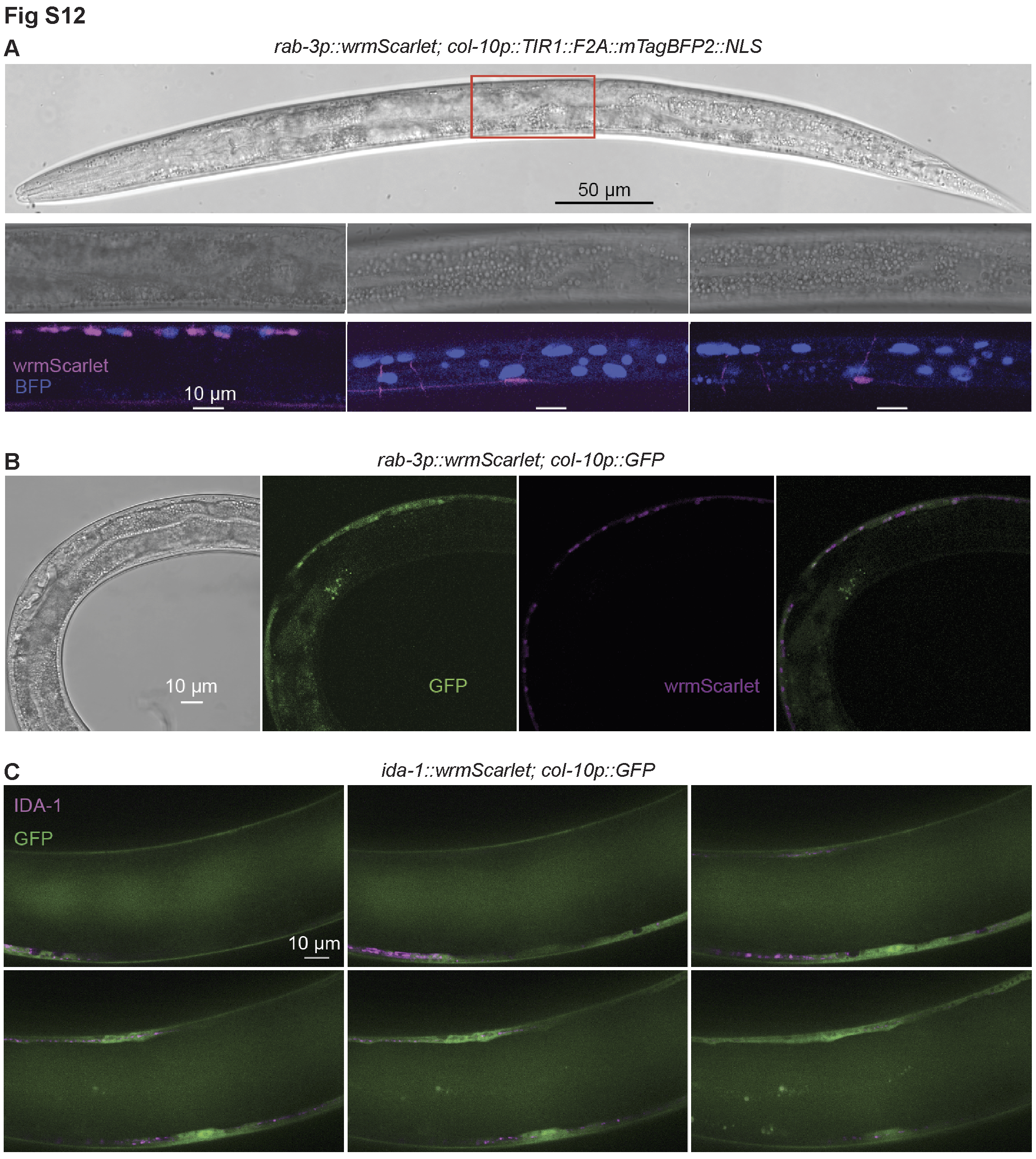

Supplement: S12 Fig — (A) The expression patterns of a DCV component gene, rab-3, and epidermal nucleus. The rab-3 promoter drove the expression of a fluorescent protein gene, wrmScarlet, indicating the expression pattern of the rab-3 transcript. Blue fluorescent protein (BFP) fused with a nuclear localization signal (NLS) was expressed in the epidermal nucleus driven by the col-10 promoter. (B) The expression patterns of rab-3 and epidermal cells labeled by a cytoplasmic GFP (col-10p::GFP). (C) Localization of endogenous IDA-1 and epidermal cells labeled with cytoplasmic green fluorescence (col-10p::GFP) in live animals. The wrmScarlet fluorescent protein gene was inserted in the C-terminus of the endogenous ida-1 gene. Magenta indicates the expression pattern of IDA-1, and green indicates epidermal cells. L3 to L4 stage animals were immobilized using 1 mM levamisole. (TIF) [file pbio.3002276.s012.tif]

Fig S2B Raw Images

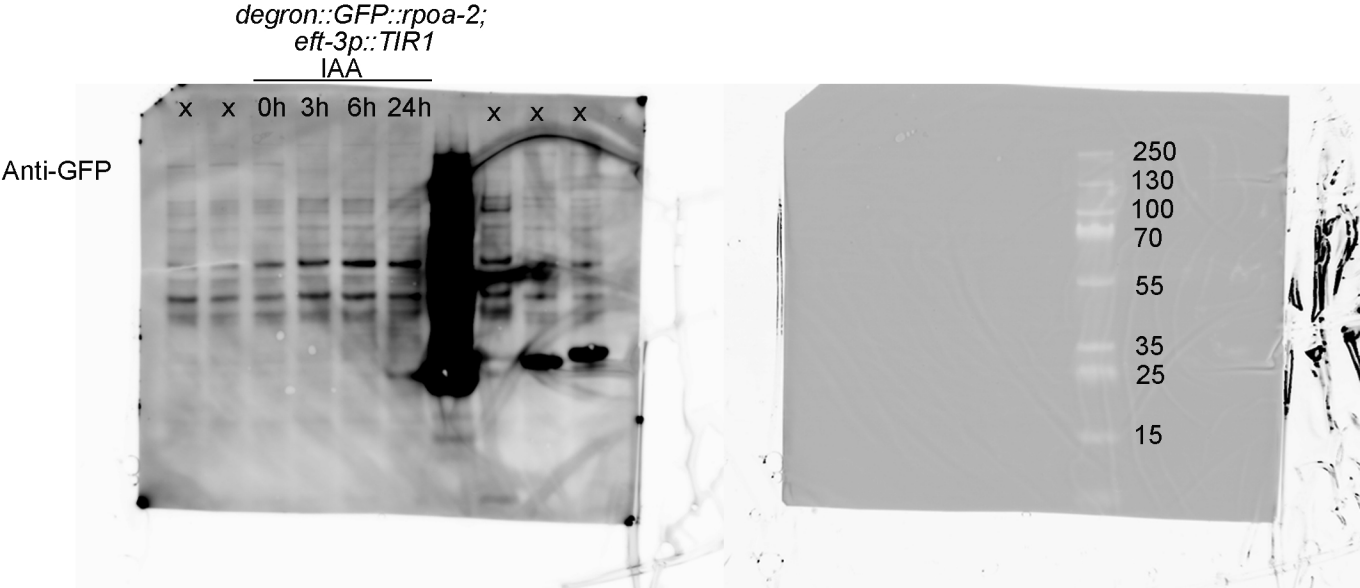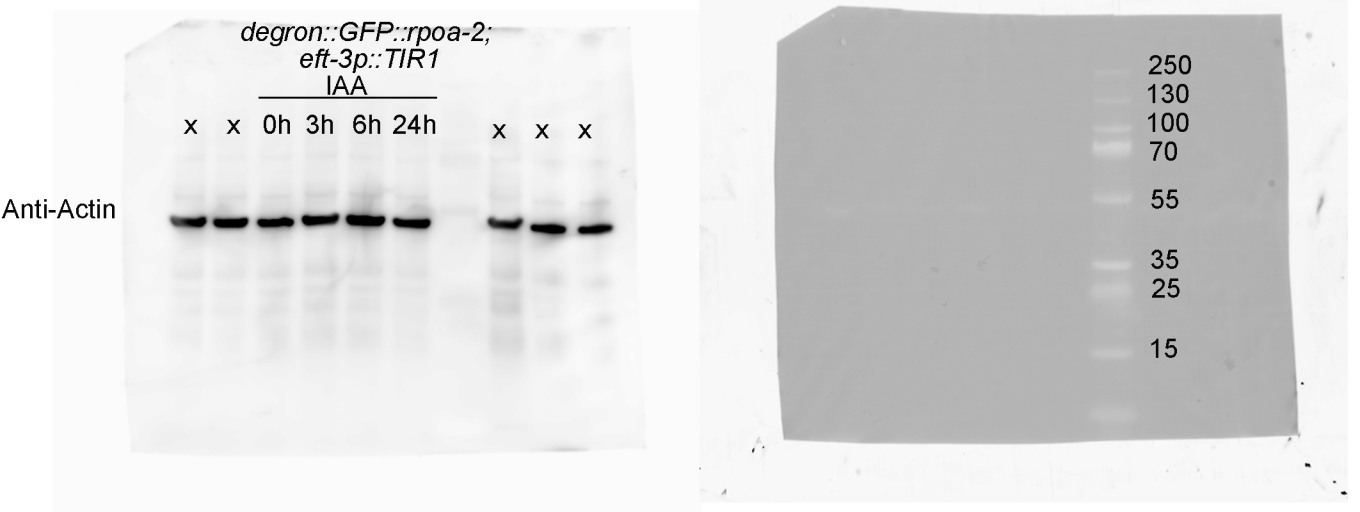

Supplement: S1 Raw Images — (PDF) [file pbio.3002276.s021.pdf]
