# Supplementary material for: Inhibition of ribosome biogenesis in the epidermis is sufficient to trigger organism-wide growth quiescence independently of nutritional status in C. elegans
Source: PLoS Biol. 2023 Aug 31;21(8):e3002276. doi: 10.1371/journal.pbio.3002276 (PMC10499265; doi:10.1371/journal.pbio.3002276)
Supplement: S4 Table — (DOCX) [file pbio.3002276.s016.docx]

**Table S4. Constructs used in this study.**

| **Constructs** | **Information** |
| --- | --- |
| pDD282 | *GFP-c1^sec^3xflag_ccdb*, Daniel Dickinson, University of Texas at Austin |
| pQZ38 | *degron-GFP-c1^sec^3xflag_ccdb*, generated from pDD282 |
| pQZ43 | *degron-GFP-c1^sec^3xflag_ccdb* edited with *rpoa-2* homologous arm inserts, generated from pQZ38 |
| pQZ69 | *degron-GFP-c1^sec^3xflag_ccdb* edited with *tsr-2(Y51H4A.15.1)* homologous arm inserts, generated from pQZ38 |
| pQZ83 | *degron-GFP-c1^sec^3xflag_ccdb* edited with *grwd-1(Y54H5A.1)* homologous arm inserts, generated from pQZ38 |
| pRB1017 | empty vector for gRNA cloning, Andrew Fire, Stanford University |
| pRR13 | *rpoa-2* sgRNA, generated from pRB1017 |
| pQZ66 | *tsr-2(Y51H4A.15.1)* sgRNA, generated from pRB1017 |
| pQZ73 | *grwd-1(Y54H5A.1) sgRNA*, generated from pRB1017 |
| pDD162 | *eft-3p^Cas9* |
| pAP087 | *ttTi5605 SEC ccdB^2x mKate2^PH^3xHA,* Daniel Dickinson, University of Texas at Austin |
| pDD122 | *eft-3p^Cas9+U6^ttTi5605^gRNA* |
| pQZ89 | *hsp-16.41p^2x mKate2^PH^3xHA*, generated from pAP087 |
| pQZ92 | *Ida-1* sgRNA, generated from pRB1017 |
| pGLOW39 | *wrmScarlet^SEC^3xMyc,* Daniel Dickinson, University of Texas at Austin |
| pQZ94 | *ida-1^wrmScarlet^SEC^3xMyc* edited with *ida-1* homologous arm inserts, generated from pGLOW39 |
| L3785 | *myo-3p^GFP* |
| pCFJ104 | *myo-3p^mCherry^unc-54 UTR* |
