# Supplementary material for: Inhibition of ribosome biogenesis in the epidermis is sufficient to trigger organism-wide growth quiescence independently of nutritional status in C. elegans
Source: PLoS Biol. 2023 Aug 31;21(8):e3002276. doi: 10.1371/journal.pbio.3002276 (PMC10499265; doi:10.1371/journal.pbio.3002276)
Supplement: S5 Table — (DOCX) [file pbio.3002276.s017.docx]

**Table S5. *C. elegans* strains used in this study.**

| **Strains** | **Genotype** | **Method** | **Source** |
| --- | --- | --- | --- |
| N2 | *WT* | NA | CGC |
| CA1210 | *dhc-1 (ie28[dhc-1::degron::GFP]) I;*  *ieSi57 [eft-3p::TIR1::mRuby::unc-54 3'UTR+Cbr-unc-119(+)] II* | NA | CGC |
| ESC315 | *ieSi57 [eft-3p::TIR1::mRuby::unc-54 3'UTR+Cbr-unc-119(+)] II* | CA1210 cross with N2 | This study |
| DV3800 | *reSi2 [col-10p::TIR1::F2A::mTagBFP2:: NLS::AID::tbb-2 3'UTR] II* | NA | CGC |
| CA1199 | *unc-119(ed3) III; ieSi38 [sun-1p::TIR1:: mRuby::sun-1 3'UTR + Cbr-unc-119(+)] IV* | NA | CGC |
| HAL230 | *unc-119(ed3) III; emcSi71 [myo-3p::TIR1::mRuby] IV* | NA | CGC |
| PD2638 | *ieSi60 [myo-2p::TIR1::mRuby::unc-54 3'UTR + Cbr-unc-119(+)] II* | NA | CGC |
| PD2632 | *ieSi61[ges-1p::TIR1::mRuby::unc-54 3'UTR + Cbr-unc-119(+)] II; unc-119(ed3) III* | NA | CGC |
| ESC319 | *qzIs15[rpoa-2p::degron::GFP::rpoa-2] I* | Microinjection | This study |
| ESC332 | *ieSi57 [eft-3p::TIR1::mRuby::unc-54 3'UTR+Cbr-unc-119(+)] II; qzIs15[rpoa-2p:: degron::GFP::rpoa-2] I.* | ESC315 cross with ESC319 | This study |
| ESC351 | *reSi2[col-10p::TIR1::F2A::mTagBFP2:: NLS::AID::tbb-2 3'UTR] II; qzIs15[rpoa-2p::degron::GFP::rpoa-2] I.* | DV3800 cross with ESC319 | This study |
| ESC352 | *qzIs15[rpoa-2p::degron::GFP::rpoa-2] I; ieSi60 [myo-2p::TIR1::mRuby::unc-54 3'UTR + Cbr-unc-119(+)] II* | ESC319 cross with PD2638 | This study |
| ESC360 | *qzIs15[rpoa-2p::degron::GFP::rpoa-2] I ; unc-119(ed3) III; emcSi71 [myo-3p::TIR1::mRuby] IV* | ESC319 cross with HAL230 | This study |
| ESC373 | *qzIs15[rpoa-2p::degron::GFP::rpoa-2] I; ieSi61[ges-1p::TIR1::mRuby::unc-54 3'UTR + Cbr-unc-119(+)] II; unc-119(ed3) III* | ESC319 cross with PD2632 | This study |
| ESC374 | *qzIs15[rpoa-2p::degron::GFP::rpoa-2] I; unc-119(ed3) III; ieSi38 [sun-1p::TIR1:: mRuby::sun-1 3'UTR + Cbr-unc-119(+)] IV* | ESC319 cross with CA1199 | This study |
| ESC424 | *qzIs32[tsr-2(Y51H4A.15.1)::Degron::GFP] IV* | Microinjection | This study |
| ESC421 | *ieSi57 [eft-3p::TIR1::mRuby::unc-54 3'UTR+Cbr-unc-119(+)] II; qzIs32[tsr-2 (Y51H4A.15.1)::Degron::GFP] IV.* | ESC315 cross with ESC424 | This study |
| ESC440 | *reSi2 [col-10p::TIR1::F2A::mTagBFP2:: NLS::AID::tbb-2 3'UTR] II; qzIs32[tsr-2 (Y51H4A.15.1)::Degron::GFP] IV* | DV3800 cross with ESC424 | This study |
| ESC432 | *qzIs35[grwd-1(Y54H5A.1)::Degron::GFP] III* | Microinjection | This study |
| ESC423 | *ieSi57 [eft-3p::TIR1::mRuby::unc-54 3'UTR+Cbr-unc-119(+)] II; qzIs35[grwd-1(Y54H5A.1)::Degron::GFP] III* | ESC315 cross with ESC432 | This study |
| ESC444 | *reSi2[col-10p::TIR1::F2A::mTagBFP2:: NLS::AID::tbb-2 3'UTR]II; qzIs35[grwd-1 (Y54H5A.1)::Degron::GFP] III* | DV3800 cross with ESC432 | This study |
| VC2372 | *rpoa-2(ok1970) I/hT2 [bli-4(e937) let-?(q782) qIs48] (I;III)* | NA | CGC |
| PD4666 | *ayIs6 [hlh-8::GFP fusion + dpy-20(+)] X* | NA | Andrew Fire, Stanford University |
| FX30167 | *tmC18 [dpy-5(tmIs1200)] I.* | NA | CGC |
| ESC382 | *rpoa-2(ok1970)/tmC18 [dpy-5(tmIs1200)] I; ayIs6 [hlh-8::GFP fusion + dpy-20(+)] X* | Crossed by VC2372, FX30167, PD4666 | This study |
| ESC394 | *reSi2[col-10p::TIR1::F2A::mTagBFP2:: NLS::AID::tbb-2 3'UTR] II; qzIs15[rpoa-2p::degron::GFP::rpoa-2] I; ayIs6 [hlh-8::GFP fusion + dpy-20(+)] X* | ESC351 cross with PD4666 | This study |
| ESC395 | *ieSi57 [eft-3p::TIR1::mRuby::unc-54 3'UTR+Cbr-unc-119(+)] II; qzIs15[rpoa-2p::degron::GFP::rpoa-2] I; ayIs6 [hlh-8::GFP fusion + dpy-20(+)] X* | ESC332 cross with PD4666 | This study |
| RDV55 | *rdvIs1 [egl-17p::Myri-mCherry::pie-1 3'UTR + egl-17p::mig-10::YFP::unc-54 3'UTR + egl-17p::mCherry-TEV-S::his-24 + rol-6(su1006)] III* | NA | CGC |
| ESC397 | *rpoa-2(ok1970)/tmC18 [dpy-5(tmIs1200)] I; rdvIs1 [egl-17p::Myri-mCherry::pie-1 3'UTR + egl-17p::mig-10::YFP::unc-54 3'UTR + egl-17p::mCherry-TEV-S::his-24 + rol-6(su1006)] III* | ESC382 cross with RDV55 | This study |
| ESC387 | *reSi2 [col-10p::TIR1::F2A::mTagBFP2:: NLS::AID::tbb-2 3'UTR] II; qzIs15[rpoa-2p::degron::GFP::rpoa-2] I; rdvIs1 [egl-17p::Myri-mCherry::pie-1 3'UTR + egl-17p::mig-10::YFP::unc-54 3'UTR + egl-17p::mCherry-TEV-S::his-24 + rol-6(su1006)] III.* | ESC351 cross with RDV55 | This study |
| CB928 | *unc-31(e928) IV.* | NA | CGC |
| ESC505 | *unc-31(e928) IV; reSi2[col-10p::TIR1::F2A:: mTagBFP2::NLS::AID::tbb-2 3'UTR]II; qzIs35[grwd-1(Y54H5A.1)::Degron::GFP] III.* | ESC444 cross with CB928 | This study |
| PD2635 | *daf-16(mu86) I.* | NA | CGC |
| PD2643 | *daf-18(ok480) IV.* | NA | CGC |
| ESC519 | *daf-16(mu86) I; reSi2[col-10p::TIR1::F2A:: mTagBFP2::NLS::AID::tbb-2 3'UTR]II; qzIs35[grwd-1(Y54H5A.1)::Degron::GFP] III.* | ESC444 cross with PD2635 | This study |
| ESC517 | *daf-18(ok480) IV; reSi2[col-10p::TIR1::F2A:: mTagBFP2::NLS::AID::tbb-2 3'UTR]II; qzIs35[grwd-1(Y54H5A.1)::Degron::GFP] III.* | ESC444 cross with PD2643 | This study |
| QK52 | *rde-1(ne219) V; xkIs99 [wrt-2p::rde-1::unc-54 3'UTR].* | NA | CGC |
| TU3401 | *sid-1(pk3321) V; uIs69 [pCFJ90 (myo-2p::mCherry) + unc-119p::sid-1] V.* | NA | CGC |
| WM118 | *rde-1(ne300) V; neIs9 [myo-3::HA::RDE-1 + rol-6(su1006)] X.* | NA | CGC |
| ESC541 | *reSi2[col-10p::TIR1::F2A::mTagBFP2:: NLS::AID::tbb-2 3'UTR] (II:0.77); qzIs15[rpoa-2p::degron(TIR1)::GFP::rpoa-2] I; rde-1(ne219) V; xkIs99 [wrt-2p::rde-1::unc-54 3'UTR].* | ESC351 cross with QK52 | This study |
| ESC540 | *reSi2[col-10p::TIR1::F2A::mTagBFP2:: NLS::AID::tbb-2 3'UTR]II; qzIs35[grwd-1(Y54H5A.1)-Degron-GFP] III; sid-1(pk3321) V; uIs69 [pCFJ90 (myo-2p::mCherry) + unc-119p::sid-1] V* | ESC444 cross with TU3401 | This study |
| ESC547 | *reSi2[col-10p::TIR1::F2A::mTagBFP2:: NLS::AID::tbb-2 3'UTR] II; qzIs35[grwd-1(Y54H5A.1):: Degron::GFP] III; rde-1(ne300) V; neIs9 [myo-3::HA::RDE-1 + rol-6(su1006)] X.* | ESC444 cross with WM118 | This study |
| DV3799 | *reSi1 [col-10p::TIR1::F2A::mTagBFP2:: AID*::NLS::tbb-2 3'UTR] I.* | NA | CGC |
| OH13908 | *daf-16(ot821[daf-16::mKate2::3xFLAG]) I.* | NA | CGC |
| ESC687 | *daf-16(ot821[daf-16::mKate2::3xFLAG]) I; reSi2[col-10p::TIR1::F2A::mTagBFP2:: NLS::AID::tbb-2 3'UTR]II; qzIs35[grwd-1(Y54H5A.1)::Degron::GFP] III* | OH13908 cross with ESC444 | This study |
| WBM1144 | *wbmIs68 [rab-3p::3XFLAG::wrmScarlet:: unc-54 3'UTR *wbmIs66] IV.* | NA | CGC |
| ESC677 | *reSi2 [col-10p::TIR1::F2A::mTagBFP2:: NLS::AID::tbb-2 3'UTR] (II:0.77); qzIs15[rpoa-2p::degron(TIR1)::GFP::rpoa-2] I; wbmIs68 [rab-3p::3XFLAG:: wrmScarlet::unc-54 3'UTR *wbmIs66] IV.* | WBM1144 cross with ESC351 | This study |
| HS445 | *dpy-22(os38) X; osEx89* | NA | CGC |
| ESC713 | *wbmIs68 [rab-3p::3XFLAG::wrmScarlet::*  *unc-54 3'UTR *wbmIs66] (IV:5015000); osEx89 [col-10::GFP + dpy-22(+)]* | WBM1144 cross with HS445 | This study |
| ESC716 | *qzIs41[ida-1:: wrmScarlet] III.* | microinjection | This study |
| ESC715 | *osEx89 [col-10::GFP + dpy-22(+)]; qzIs41[ida-1:: wrmScarlet] III.* | HS445 cross with ESC716 |  |
| DLW109 | *wrdSi23 [eft-3p::TIR1:F2A:mTagBFP:tbb2 3' UTR:: loxP] I; unc-104(knu973[unc-104::AID]) II.* | NA | CGC |
| ESC719 | *wrdSi23 [eft-3p::TIR1:F2A:mTagBFP:tbb2 3' UTR:: loxP] I; qzIs38[hsp-16.41p::mKate2] (II, ttTi5605); qzIs34[grwd-1(Y54H5A.1):: Degron::GFP] III.* | DLW109 cross with ESC717 | This study |
| ESC717 | *qzIs38[hsp-16.41p::mKate2](II, ttTi5605); qzIs34[grwd-1(Y54H5A.1)::Degron::GFP] III.* | microinjection | This study |
| ESC720 | *qzIs38[hsp-16.41p::mKate2](II, ttTi5605); qzIs34[grwd-1(Y54H5A.1)::Degron::GF)] III; reSi1 [col-10p::TIR1::F2A::mTagBFP2:: AID*::NLS::tbb-2 3'UTR] I.* | ESC717 cross with DV3799 | This study |
| ESC721 | *qzIs41[ida-1:: wrmScarlet] III; reSi2 [col-10p::TIR1::F2A::mTagBFP2:: NLS::AID::tbb-2 3'UTR] (II:0.77); qzIs15 [rpoa-2p:: degron::GFP::rpoa-2] I.* | ESC716 cross with ESC351 | This study |
