# Supplementary material for: Inhibition of ribosome biogenesis in the epidermis is sufficient to trigger organism-wide growth quiescence independently of nutritional status in C. elegans
Source: PLoS Biol. 2023 Aug 31;21(8):e3002276. doi: 10.1371/journal.pbio.3002276 (PMC10499265; doi:10.1371/journal.pbio.3002276)
Supplement: S6 Table — (DOCX) [file pbio.3002276.s018.docx]

**Table S6. Oligos used in this study.**

| **Oligos** | **Sequences** |
| --- | --- |
| AF-ESC-702 | GCATCCCCTAAAGATCCAGCCAAACCTCCGGCCAAGGCACAAGTTGTGGGATGGCCACCGGTGAGATCATACCGGAAGAACGTGATGGTTTCCTGCCAAAAATCAAGCGGTGGCCCGGAGGCGGCGGCGTTCGTGAAGggatccgga |
| ESC-RR-1 | ACGTTGTAAAACGACGGCCAGTCGCCGGCACACTCGCATTTAGGCGGGAA |
| ESC-RR-3 | CGTGATTACAAGGATGACGATGACAAGAGAATGGACTGCGACATAGCGTC |
| ESC-RR-4 | TCACACAGGAAACAGCTATGACCATGTTATGGCAACCTTCGGATCGGAGC |
| ESC-RR-5 | TCT TGC TTT CAA GTT TTC AGG CGA |
| ESC-RR-6 | AAA CTC GCC TGA AAA CTT GAA AGC |
| ESC-QZ-143 | AGGTTTGGCTGGATCTTTAGGGGATGCCATcgcctgaaaacttgaaagttt |
| ESC-QZ-266 | TCTTGcaaacagggagcaataaca |
| ESC-QZ-267 | AAACtgttattgctccctgtttgC |
| ESC-QZ-270 | acgttgtaaaacgacggccagtcgccggcaGGCTGTTGGAGCTAATCCAT |
| ESC-QZ-271 | GAGGCTCCCGATGCTCCAATATTAATAGTCTTGAAGACATTAAATCCATC |
| ESC-QZ-272 | CAAGGATGACGATGACAAGAGATAAGATGTCTCCCTGTTATTGCTCC |
| ESC-QZ-273 | cagctatgaccatgttatcgatttGGTCTCGGTAGGTATTGGCG |
| ESC-QZ-233 | TCTTGTGTTGTAGACGATGATGGC |
| ESC-QZ-234 | AAACGCCATCATCGTCTACAACAC |
| ESC-QZ-237 | acgttgtaaaacgacggccagtcgccggcaGTAAATATGAGCATAAATGCCGACG |
| ESC-QZ-238 | AGGTTTGGCTGGATCTTTAGGGGATGCCATTTGTCTTTTGGTGATTGTCGTCC |
| ESC-QZ-239 | CGTGATTACAAGGATGACGATGACAAGAGATAAACTTTATTGATTTTTTTTTCAAAATAT |
| ESC-QZ-240 | tcacacaggaaacagctatgaccatgttatCGTCTCATTTTGGAGGGAAT |
| ESC-QZ-363 | CATCCtgtaaaacgacggccagtgcGGCCGCgatcaccaaaaacggaacg |
| ESC-QZ-364 | CATGTTTTCTTTAATGAGCTCGGAGACCATtttcgaagttttttagatgc |
| ESC-QZ-374 | TCTTGaactctctcttttgtagtg |
| ESC-QZ-375 | AAACcactacaaaagagagagttC |
| ESC-QZ-378 | CAATTTCACACAGGAAACAGCTATGACCATGTTGATGGCTCAGGTCGTACAGG |
| ESC-QZ-379 | CCTCTCCCTTGGAGACCATtccggatcctccTTTAGTAGCGATTGACTTTAGAAGATGGT |
| ESC-QZ-380 | GTTGATCAGCGAGGAAGACTTTAAttaaaatttTACGCAATCGTTGTTCCCCA |
| ESC-QZ-381 | CCCAGTCACGACGTTGTAAAACGACGGCCAGTCCCTCAAACAACCAGCTGAACG |
