# Supplementary material for: Inhibition of ribosome biogenesis in the epidermis is sufficient to trigger organism-wide growth quiescence independently of nutritional status in C. elegans
Source: PLoS Biol. 2023 Aug 31;21(8):e3002276. doi: 10.1371/journal.pbio.3002276 (PMC10499265; doi:10.1371/journal.pbio.3002276)
Supplement: Attachment — Submitted filename: Zhao et al. response to reviewers.pdf [file pbio.3002276.s022.pdf]

RESPONSE TO REVIEWS:

Note: We have replaced the term 'hypodermis' with 'epidermis' throughout the document. This change was suggested by Reviewer 3 and is based on the fact that the hypodermal cells in *C. elegans* are functionally equivalent to the tissue known as the 'epidermis' in other organisms. (Chisholm & Hisao 2012, *PMID: 23539299*).

Reviewer #1: In this manuscript, Zhao and coworkers identified ribosome biogenesis inhibition as a novel cell non-autonomous growth checkpoint in C. elegans. They found that auxin-inducible degradation of the RNA Pol I subunit rpoa-2 or two chaperones for ribosomal proteins (rrb-1 and tsr-2) in whole animals leads to an L2 larval arrest and shows similar gene expression signatures as UV-exposure. Moreover, there are also partial overlaps with dauer and starvation-induced L1 arrest, as well as with DAF-16 target genes. Hypodermis-specific depletion of RPOA-2 phenocopies whole body depletion, suggesting that loss of ribosome biogenesis in the hypodermis elicits an organism-wide response. Finally, the authors found that RNAi-mediated knockdown of unc-31 in the whole body or specifically in the hypodermis partially rescued the growth quiescence upon hypodermal RPOA-2 depletion.
Overall, the study by Zhao and coworkers is highly interesting to a broad readership and, therefore, suitable for PLoS Biology. The methods are state-of-the-art, especially the AID system and the comprehensive bioinformatic analysis. The newly generated degron strains were convincingly validated and represent a valuable tool for the community. The manuscript is well written, and data are presented clearly in all Figures, Supplementary Figures, and Tables.
The major drawback of the study is that direct experimental proof for cross-talk from the hypodermis to other tissues is missing, apart from the modest rescue of growth arrest by unc-31 RNAi and indirect evidence from bioinformatics.

We appreciate the thoughtful review and the positive assessment of our manuscript's interest to a broad readership. To address the identified drawback of missing experimental validation for cross-talk from the hypodermis to other tissues, we carried out several additional experiments.

First, we have found that the inhibition of hypodermal (epidermal) ribosome biogenesis reduces new protein synthesis in other tissues. This finding is supported by our use of an inducible heat shock reporter (*hsp-16.41p::mKate2*) (**Revised Fig 6A,** page 12 lines 21-29).

Second, we have identified IDA-1 as a target for cell non-autonomous gene expression changes. We found that the IDA-1 protein is predominantly overexpressed in pharyngeal neurons in response to the inhibition of ribosome biogenesis in the hypodermis (epidermis) (**New Fig 8C,** page 16 lines 17-18).

Finally, we have discovered that the hypodermis (epidermis)-specific knockdown of IDA-1, which is another component of the DCV pathway, can suppress the organism-wide growth

quiescence triggered by the inhibition of hypodermal (epidermal) ribosome biogenesis(**New Fig 8A,** _page 16 lines 6-10_).

Taken together, these findings provide evidence for the existence of cross-talk from the hypodermis to other tissues. In response to the other points raised, we have provided a point-by-point response below.

Please find my specific comments below.

Major concerns:
1.) The whole study is based on the assumption that RPOA-2 depletion by AID inhibits ribosome biogenesis in whole nematodes or, specifically, in the hypodermis. This should be experimentally verified by demonstrating that fewer ribosomes are present, e.g., by quantifying 25S and 18S rRNA levels.

We appreciate the reviewer's suggestion. To verify our assumption that RPOA-2 depletion by AID results in fewer ribosomes, we performed sucrose gradient ultracentrifugation to examine the quantity of ribosomes present. We observed a notable decrease in both small and large subunits, monosomes, and polysomes following RPOA-2 depletion (**Revised Fig S2C,** _from page 5 line 26 to page 6 line 7_). These findings provide support for our hypothesis that RPOA-2 depletion leads to a reduction in ribosome biogenesis.

2.) Ribosome biogenesis inhibition will likely cause a reduction of global protein synthesis. The authors should verify this, e.g., by radioactive or puromycin labeling. Moreover, a recent study introduced a novel protocol to analyze protein synthesis in a tissue-specific manner (https://doi.org/10.1016%2Fj.crmeth.2022.100203). The authors could utilize this method to test if the degradation of RPOA-2 in the hypodermis also affects protein synthesis in other tissues.

We appreciate the reviewer's suggestion to verify the reduction of global protein synthesis upon ribosome biogenesis inhibition and to assess if the degradation of RPOA-2 in the hypodermis (epidermis) affects protein synthesis in other tissues.

We have thoroughly considered the use of the O-propargyl-puromycin labeling method recently published (Somers et al. 2002, *PMID: 35497499*). While the technique indeed offers tissue-specific analysis of protein synthesis, we were concerned about the potential bias introduced by the permeation of puromycin and the label through the cuticle, particularly as our proteomic analysis revealed a decrease in cuticle genes when hypodermal (epidermal) ribosome biogenesis is inhibited.

To circumvent this issue, we chose an alternative approach to assess global protein synthesis using an inducible heat shock reporter (*hsp-16.41p::mKate2*). We constructed a strain with a single copy insertion of the inducible reporter, driven by a heat shock promoter (*hsp-16.41p*) and encoding a red fluorescent protein (mKate2) on Chr II. We then crossed this reporter strain

with global and hypodermal (epidermal) inducible ribosome biogenesis inhibition strains (*rrb-1::degron::GFP III*; *eft-3p::TIR1 I* or *col-10p::TIR1 I*, respectively).

Following a 3-hour heat shock, we observed a significant reduction in mKate2 production in both the global and hypodermal (epidermal) ribosome biogenesis inhibition strains compared to controls (**Revised Fig 6A**). Given that the majority of the *hsp-16.41p::mKate2* expression can be detected in non-hypodermis (epidermis) cell types, this data provides evidence that inhibition of ribosome synthesis in the hypodermis (epidermis) leads to a decrease in new protein synthesis in other tissues. We have included these results in our revised manuscript (*page 12 lines 21-29*).

3.) To complete the validation of their experimental model, the authors should verify in degron::GFP animals (lacking TIR1) that IAA exposure alone does not cause a growth phenotype.

Thanks for the suggestion, we confirmed that IAA exposure alone does not induce a growth phenotype in degron::GFP animals lacking TIR1 (figure pasted below).

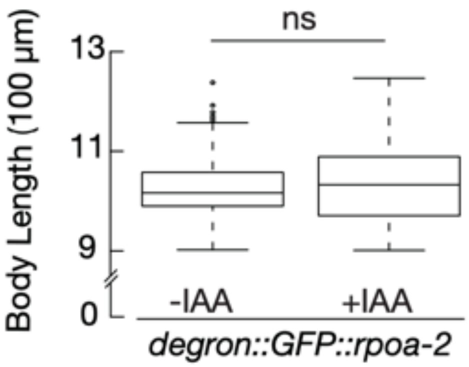

Figure: Exposure to 1mM IAA does not lead to growth defects in the worms. The body length of the worms were measured from three independent experiments with or without IAA, each containing at least 25 worms per replicate. 'ns' indicates a p-value greater than 0.05 as determined by an independent t-test.

4.) Although the inhibition of ribosome biogenesis at different steps by the AID system is elegant and specific, it would still be interesting to see if chemical inhibition of Pol I transcription, e.g., by CX-5461 or Actinomycin D, causes a similar growth quiescence phenotype.

We value the reviewer's suggestion to assess the effects of chemical inhibition of Pol-I transcription with compounds like CX-5461 or Actinomycin D. In our work, we effectively leveraged the AID system to degrade RPOA-2, which in turn inhibited RNA Pol-I transcription (**Fig 1C, Revised S2B, S2C,** *from page 5 line 26 to page 6 line 7*). While the application of chemical inhibitors is an intriguing alternative strategy, it's crucial to note that CX-5461 and Actinomycin D have reported impacts on Top2 and RNA Pol-II respectively (Pan et al. 2021, *PMID: 34753908*; Casse et al. 1999, *PMID: 10347161*). These additional effects introduce complexity to their use in our context, as any resultant effects may not be exclusively due to Pol-I inhibition.

While we appreciate the potential enrichment these experiments could provide to our study, considering these factors, we respectfully suggest that such tests fall outside the scope of our current investigation.

5.) The conclusion that daf-16 is repressed upon RPOA-2 depletion should be experimentally verified.

To address this concern, we conducted the following experiments:

We crossed a CRISPR-engineered *daf-16* allele, integrated with *mKate2* at the C-terminus, and the inducible strain for hypodermal (epidermal) ribosome biogenesis inhibition (*rrb-1::degron::GFP; col-10p::TIR1*). Under standard conditions, we detected no nuclear localization of DAF-16::mKate2 in L1 larvae, regardless of whether ribosome biogenesis in the epidermis was inhibited (+IAA) or not (-IAA). DAF-16 should primarily be observed in the nucleus when activated (e.g., during starvation). Hence, our result suggests that DAF-16 was not activated in these contexts (**Revised Fig S7D**). Upon subjecting these animals to starvation, we observed nuclear localization of DAF-16::mKate2 (**Revised Fig S7E**). However, during starvation, nuclear DAF-16::mKate2 density decreased when hypodermal (epidermal) ribosome biogenesis was inhibited (+IAA) compared to the control (-IAA) (**Revised Fig S7E**). This implies a potential repression of DAF-16 activation during the inhibition of hypodermal (epidermal) ribosome biogenesis (<u>*from page 10 line 29 to page 11 line 14*</u>).

We've updated our manuscript to clarify that our findings suggest a lack of DAF-16 activation, rather than its repression, in the context of RPOA-2 depletion. An earlier result section also now reads " *…suggested that DAF-16 is likely not activated during RPOA-2 depletion"* (**Fig S5B, S5C;** *page 9, line 4).*" We thank the reviewer for suggesting these experiments that we believe further clarifies our original conclusion.

6.) Why were the worms raised at 16°C for some experiments (e.g., Figure 2C) and 20°C for others (e.g., 4D)? Please confirm that the temperature only affects the timing but not the larval stage of growth arrest.

The chosen temperatures were based on the natural growth rates of *C. elegans* at these temperatures, which allowed us to observe the impact of ribosome biogenesis inhibition at various rates of larval development.

Our findings show that while temperature influences the rate of development, it does not affect the stage of growth arrest triggered by ribosome biogenesis inhibition. Even though larvae tend to develop more rapidly at 20 °C compared to 16 °C under normal conditions, we observed that growth quiescence occurred at the same larval stage in both scenarios, despite the different temperatures.

This evidence suggests that temperature changes primarily affect the timing but not the stage of growth quiescence induced by ribosome biogenesis inhibition. We have now included this

clarification in the revised manuscript (<u>*page 10 line 16 & page 24 lines 22-25*</u>) to address the reviewer's concerns.

Minor comments:
1.) It is not clear from how many independent experiments the indicated numbers of animals are derived.

We have addressed this concern by including the necessary details in the figure legends (**Fig 1, 2, 4, 7, 8, S3, S6, S7, S11**). The figure legends now specify the number of biological replicates and the total number of animals included in the quantification. We want to clarify that the graphs display the quantification of all data points, unless specifically mentioned otherwise. The animal numbers are summed from all three biological replicates to provide a comprehensive representation of the results.

2.) T-tests without corrections for multiple comparisons are unsuitable for comparing more than two groups (e.g., Figures 4A-C). Please include a correction for multiple comparisons or perform ANOVA with post hoc tests instead.

We have now performed a post-hoc Bonferroni correction to all p-values resulting from multiple comparisons (**Fig S3A, 4A-C, S7A, S7B, 8A, 8D**). After applying this correction, our original conclusions remain unchanged. We have updated the manuscript to reflect this change in statistical analysis and the corresponding results.

3.) Please include more details on the description of the bioinformatic methods. Were p-value, FC, or base-mean cutoffs used for the GO-term analysis? For the bioinformatic comparisons with published datasets, were the raw data re-analyzed using the same pipeline as the new data generated here, or were the already available gene lists used for the comparisons? Please also specify the original method (RNA-seq or microarray) and which sample groups of the published data were used for the comparisons.

We appreciate the reviewer's request for additional details on our bioinformatics methods.

For the GO term analysis, we utilized significantly overexpressed or underexpressed genes as inputs to FuncAssociate (Berriz et al., 2009). All detected genes were considered as the background. FuncAssociate uses Fisher's Exact test to calculate the association p-value between the GO attribute and the query genes, adjusted by for multiple testing. All GO categories with significant adjusted p-values from our RNAseq and proteomic analyses are detailed in **Tables S1-S3**, which include the corresponding log odds ratios (LOD). Some redundancy may occur due to overlapping or encompassing GO categories, and to simplify representation, we selected representative categories for plots in **Fig 5C, 6D**.

For RNAseq comparisons in Figure 3, we used gene sets identified in two studies (Stadler et al., 2013 *PMID: 24098135*; Cenik et al., 2019 *PMID: 30799226*) related to ribosome large subunit deficiency and starvation. We compared these sets to those affected by RNA Pol I depletion,

using Fisher's exact test to calculate log-odds ratios and statistical significance (**Fig 3C, 3D**). For dauer conditions, we used a gene set from a microarray analysis (McElwee et al., 2006, *PMID: 16522328*), using Fisher's exact test for comparisons (**Fig 3E**). In Figure S5, we used gene sets identified in studies by Kumar et al. (2015, *PMID: 26539642*) and Mueller et al. (2014, *PMID: 25419847*), comparing these to genes affected by RNA Pol-I depletion and conducting Fisher's exact test for statistics (**Fig S5C, S5E**). We have now expanded upon these procedures in the methods section to ensure our approach is clear (*from page 28 line 16 to page 29 line 12)*. We thank the reviewer for suggesting this clarification.

4.) The sentence "The growth checkpoint…" (p3 line 7) is duplicated in p3 line 13.
5.) The last paragraph of the introduction (p3 line 18) is highly redundant (almost literal) with the abstract and could be shortened.
6.) Figure 4D: It is unclear what "L1" at the top refers to. It might be useful for readers to include "L1", "L2" and "L3" in the corresponding figure panels, like in Figure 2C.
7.) The introduction and discussion sections still contain minor typos and language errors.
8.) p15 line 25ff and p16 line 8ff are redundant ("Why does the hypodermis affect…").

We sincerely appreciate the reviewer's thorough examination of the manuscript. We have revised the text accordingly. We rewritten the section related to point 4 (*page 3 lines 12-21)* and point 5 (*from page 3 line 22 to page 4 line 30*).

Reviewer #2: The manuscript submitted by Zhao et al. is a continuation of an interesting study published by Cenik et al. in Developmental Cell in 2019, wherein the authors showed that maternal ribosomes are sufficient to carry out all aspects of embryonic development and that only when specific contexts are met during postembryonic development do animals arrest in the absence of newly synthesized ribosomes. In addition to this important information, these authors carried out an interesting series of mosaic analyses that allowed them to conclude that growth was mediated through some non-autonomous regulation between cells in the growing larva.

This new work attempts to build on this interesting observation by trying to identify the tissues involved in this regulation and the molecules that might contribute to the growth arrest associated with a lack of ribosomes or ribosomal function.

The authors use AIDs to genetically dissect the contributions of various tissues in this process in addition to extensive transcriptome and proteomic analysis to compare the changes in the ribosome-compromised animals. Although extensive, the authors finally focus on the role of a well known gene product that is involved in neuronal secretion of vesicles, which they eventually show may be playing a role in the hypodermis in order to mediate the ribosome-compromised signal to the rest of the animal to block growth and ensure appropriate scaling of the organism. This aspect (how an animal scales growth) of the manuscript is quite novel but is not fully elaborated beyond the observed organismal growth arrest that is induced following growth compromise in the hypodermis due to ribosomal dysfunction.

Overall, the manuscript is reasonably well written, although there are sections that are confusing and the rationale is not entirely clear. On the other hand the experiments are technically well performed and the data are solid and well controlled. The interpretations are also reasonable and are based on appropriate statistical analysis. The manuscript includes enormous amounts of data that have little impact on the most interesting aspect of the work, that being the basis of the non-autonomous effect of tissues to ensure growth arrest and appropriate scaling.

The final series of experiments with unc-31 take the investigators incrementally forward in understanding how this non-autonomous signal is mediated. However, one could have intuitively predicted, or at least test this possibility, prior to engaging in the multiple omics-style analyses that contribute little to the final conclusions that the authors make. They do tell us that metabolic compromise is not the same as compromise of protein synthesis, which seems obvious for anybody that works on metabolism, but comparing all these omic signatures with other states where starvation associated quiescence is involved does provide some novel information. Unfortunately, the few proteomic hits that encourage the authors to proceed toward examining a role for secreted factors are mentioned, but not followed up or validated, making the entire contribution of these studies to the final conclusions pretty minor.

We appreciate the reviewer's comprehensive overview of our study and their recognition of the novelty and technical quality of our work.

Responding to their points on the non-autonomous effect of tissues in ensuring growth arrest and appropriate scaling, we have conducted experiments to further strengthen our hypothesis. New evidence on the effects of hypodermis (epidermis)-specific ribosome biogenesis inhibition on decreasing protein synthesis across the organism is now included (**Revised Fig 6A,** *page 12 lines 21-29*). We have also found that IDA-1, a membrane-associated protein of the dense core vesicle (DCV), is present in or nearby hypodermal (epidermal) cells, and its hypodermis (epidermis) specific knockdown suppresses organism-wide growth quiescence in response to hypodermal ribosome synthesis inhibition (**New Fig 8A, 8B, Table S3,** *page 16 lines 6-20*). We further observed increased DCV puncta, labeled by IDA-1, when hypodermal (epidermal) ribosome biogenesis was inhibited, indicating that the non-autonomous signal is potentially mediated by the DCV pathway (**New Fig 8B, 8C,** *page 16 lines 6-20*).

We have followed the reviewer's suggestion to follow up on the hits from our proteomics analysis. Nine hits that were overexpressed, secreted, and localized to the hypodermis (epidermis) have been investigated further (CPR-4, LBP-1, LBP-2, FAR-1, NPA-1, CPR-1, MEC-5, TTR-5, TTR-2). We found that reducing the expression of four of these –lbp-2, far-1, cpr-1, ttr-2– in hypodermis (epidermis) significantly increased body size in response to hypodermal (epidermal) ribosome biogenesis inhibition (**New Fig 8D,** *page 16 lines 21-28*). Of these candidates, only FAR-1 had an effect size on body length comparable to UNC-31 (*from page 16 line 29 to page 17 line 9*).

Given that the knockdown of these four genes did not fully recover the growth phenotype associated with hypodermal(epidermal) ribosome biogenesis inhibition, we hypothesize additional, yet unidentified, components also play a role in this non-autonomous signaling. In our future research, we intend to address this possibility and characterize the potential signaling pathways involved in this process.

Lastly, we acknowledge the reviewer's critique regarding the omics analyses. However, in addition to being comprehensive, they have provided important directions to explore. As appreciated by all three reviewers, the current work represents a major effort towards understanding the non-autonomous effect of tissues to ensure growth and organ scaling. While we have not been able to follow up all the exciting directions that emerged from our omics-style profiling, we believe that these data provide fertile ground for future experimentation by us and others in the field.

I have listed a few concerns that I find problematic with the manuscript.
The authors claim that the cell non-autonomous signal originates in the ribosome-compromised hypodermis to regulate growth in non-compromised tissues. This may be overinterpeted since, as indicated in Figure 4, most tissues show some effect on limiting growth when they are compromised for ribosomal function. This could mean that all tissues are capable of this form of regulation to scale the organism, but because the

hypodermis is one of the largest organs, its role is the most obvious. It is therefore possible that unc-31 may be playing a similar role in all the other tissues, but their individual contributions may simply be more difficult to quantify since it would be inherently less than that of the comparatively much larger hypodermis. This might also be the basis of the difference that is noted between a loss of ribosome function in the hypodermis which results in an L3 arrest vs total loss of ribosomal function which causes an L2 arrest.

We thank the reviewer for suggesting the hypothesis that organ size could be a determinant of the observed phenotype. To evaluate this, we referenced the tissue volume measurements provided by Froehlich et al., 2021, *PMID: 33426507*, Fig 1). Our analysis reveals that the overall volume of the hypodermis (epidermis) is similar to the other organs we studied, including the body wall muscle, intestine, pharynx, and gonad. Yet, our results showed that animals with ribosome biogenesis inhibited in the body wall muscle, intestine, pharynx, and gonad could progress to the L4 stage (**Fig 4**), while animals with hypodermal (epidermal) ribosome biogenesis inhibition remained at the L3 stage even after 5 days (**Fig 4, S7C**). These results highlight the unique significance of the hypodermis (epidermis) in growth regulation, which seems to extend beyond its proportional size.

We agree with the reviewer that UNC-31 may indeed play similar roles in other tissues. In light of this feedback, we have modified our discussion to present a more nuanced interpretation of our results (*from page 20 line 29 to page 21 line 7*). We now emphasize that while our findings highlight the critical role of the hypodermis (epidermis) in coordinating organism-wide growth, there may well be contributions from other tissues as well.

These data incrementally advance what the corresponding author demonstrated in 2019 with a Dev Cell paper. The key question that I assume they are interested in answering is how this cell non-autonomy is generated. If the authors performed a genetic screen to identify unc-31 as a suppressor of the M cell division regulation, they must have also found the secreted target that is released by UNC-31 to mediate the regulation. This information is critical and would embellish this current work/submission.

We appreciate the comment and agree that understanding the mechanism of cell non-autonomy is a critical aspect of our work. To address the reviewer's point, in the revised manuscript, we have further analyzed nine candidates that are overexpressed, secreted, and localized in the hypodermis (or epidermis) (CPR-4, LBP-1, LBP-2, FAR-1, NPA-1, CPR-1, MEC-5, TTR-5, TTR-2). Among the candidates we investigated, we discovered that when *far-1* was knocked down in the epidermis, it mitigated the growth stagnation due to epidermis-specific ribosome biogenesis inhibition to a similar degree as *unc-31*. The FAR-1 protein belongs to the FAR family, which are small, helix-rich, and secreted proteins that bind to fatty acids and retinol (**New Fig 8D**, *from page 16 line 21 to page 17 line 9*). This initial exploration of secreted factors broadens our understanding of cell non-autonomous regulation and highlights new avenues of investigation in future work.

The limited effect of the unc-31 RNAi on suppressing organismal growth when eliminated in the hypodermis is accounted for by indicating that the RNAi might not be efficient or that there is redundancy. The unc-31 RNAi efficiency in the hypodermis can be controlled to test this idea, but alternatively, it could be due to unc-31 function in other tissues that contribute to the non-autonomous growth regulation. This needs to be elaborated and/or more rigorously investigated.

We appreciate the reviewer's feedback. To address concerns about RNAi efficiency, we conducted alternative experiments in addition to tissue-specific strains fed with RNAi bacteria (**Fig 7**). Specifically, we performed RNAi through the injection of DNA that produces double-stranded RNA targeting hypodermal (epidermal) *unc-31* (*wrt-2p::unc-31*, **New Fig S11B**), and by immersing hypodermis (epidermis) RNAi sensitive larvae in a double stranded RNA solution (**New Fig S11C,** *page 15 lines 21-24*). These approaches led to similar suppression of organismal growth (median ~6%, epidermal RNAi, soaking method). This further underscores the role of UNC-31 in the hypodermis (epidermis) in regulating growth in response to hypodermal (epidermal) ribosome biogenesis inhibition.

To address the function of *unc-31* in other tissues, we performed knockdown experiments in neurons and body-wall muscle, and observed varied phenotypes (**Fig 7D, 7E**). This implies the complex nature of UNC-31's role across different tissues, which may partly explain the modest effects observed upon its hypodermal (epidermal) knockdown.

In response to the feedback, we have revised the discussion section to acknowledge the potential contribution of UNC-31 function in neurons located beneath the hypodermal (epidermal) cells in overall growth inhibition (*from page 20 line 29 to page 21 line 4*).

I am not sure that this regulation can be referred to as a checkpoint, since there is no obvious contingency that needs to be met to advance in the growth pathway. However, the idea of tissue scaling is intriguing and that tissues need to communicate in order to grow together and not in an uncoordinated manner. Maybe the focus of this work could be adjusted to address this developmental problem without invoking some growth checkpoint that does not have an obvious trigger.

We are grateful for the reviewer's recommendation. In response, we have now emphasized the role of the epidermis (hypodermis) in scaling organism-wide growth, with due attention given to other tested organs–body wall muscle, intestine, pharynx, and germline– each contributing to different degrees.

We have also emphasized our findings of a reversible, stage-specific slowdown in growth (instead of referring this as a checkpoint), which we call quiescence, at the L2 and L3 stages (**Fig 3 and 4, examples:** *page 8 line 3, page 10 line 8*), in contrast to the unimpeded progression into adulthood observed when ribosome synthesis is inhibited at the L4 stage (**Fig S4A**). We have expanded upon these points in a more detailed manner within the discussion section of our manuscript (*page 18, lines 8-22*). This refinement better aligns our study with the

developmental dilemma emphasized by the reviewer, thus eliminating the need to invoke a growth checkpoint concept.

The proteomic targets that are cherry-picked from the data include secreted proteins that the authors suggest are indicative of some secretory pathway being involved. The target ENDU-2 is indeed a secreted endoribonuclease that culls specific somatic RNAs from the germ cells. It is not clear what this protein would be doing in this context, but if it is indeed present in higher levels, then it should leave some kind of change in the steady state RNA footprint, which might be relevant for this phenomenon. However, the authors mention it as a means of corroborating their secretion idea, and do not do any further experiments to follow up on its involvement. (2.2) What about CPR or TTR gene products? Why were these not validated for their implication in this process? This leads the reader to surmise then that they simply aren't involved, which they might not be, and therefore makes all the other conclusions about this proteomic analysis and its relationship to the authors' claims somewhat questionable.

We greatly appreciate the reviewer's insight and acknowledge the need to further investigate the roles of the secreted proteins identified in our study. With regards to ENDU-2, we agree that its specific function in this context remains uncertain, and given its underexpression (**Fig 6C**), we chose not to delve deeper into this particular candidate.

In response to the comments, we have now conducted additional experiments, specifically focusing on nine overexpressed, secreted, and hypodermally (epidermally) localized candidates: CPR-4, LBP-1, LBP-2, FAR-1, NPA-1, CPR-1, MEC-5, TTR-5, and TTR-2. After reducing the expression of one of these candidates (*far-1*) in the hypodermal (epidermal) tissue, we noticed a significant and considerable increase in body size upon hypodermal (epidermal) ribosome biogenesis inhibition (**New Fig 8D,** *page 16 line 21 to page 17 line 8*). In nematodes, the *far-1* mRNA is specifically expressed in the epidermis (Cheng et al. 2013, *PMID: 23755297* ; Ding et al. 2019, *PMID: 34576221*). The epidermal expression of *far-1* and its subsequent reduction in epidermis resulting in a significant increase in body length, suggests its potential role as a growth inhibitor during epidermal ribosome biogenesis inhibition.

We think that these additional analyses provide a more comprehensive understanding of the potential secreted factors and significantly strengthen our manuscript.

I also noted number of minor issues which I have listed below:
Page 9 Lines 5/6- this is awkward. Seems to be missing "is" or should read "differs"
Page 15 Line 2- ; the mesoblast....and the vulval precursor cells...
Page 8 Line 13- r in rad should be italicized
Page 29 Line 10- The language here seems incorrect. UNC-31 does not promote growth in my opinion. It is passive and is necessary for food intake which in turn may affect regulators that promote growth. If you introduce UNC-31 into a tissue that normally does not grow it will not suddenly grow because UNC-31 is present. Therefore, it permits

growth due to its role in neuromuscular control of foraging behaviours and maybe even pharyngeal pumping.

Page 29 The entire section pertaining to the neuronal investigation of unc-31 and its role in this phenomenon is a bit confusing to read. Setting out the rationale of these experiments in a clearer way would make the section easier to understand and interpret.

We are grateful for the reviewer's feedback which helped enhance the clarity of our manuscript. We have now revised these sentences.

Page 29 Line 10 - We agree with the reviewer's suggestion and have revised the sentence to clarify the role of UNC-31 in the results section. It now reads, "..UNC-31's role in neuromuscular control of foraging behaviors, expressed in neurons and body wall muscles, might be essential for enabling adequate feeding and consequently promoting growth (*page 15 lines 7-10*)"

Page 29 - We have now rewritten this entire section (*from page 14 line 28 to page 15 line 27*) to provide clearer rationales for the experiments and improve the overall interpretability.

Reviewer #3: This manuscript uses tissue specific inhibition of C. elegans orthologs of ribosome assembly factors to show that hypodermal (epidermal) ribosome function is required for overall growth of the animal. This work follows up previous studies on genetic mosaics for ribosome synthesis that show a developmental arrest; it extends the analysis to inhibit ribosome assembly in specific tissues and examines the effects on global gene expression and proteomes. The authors find expression of many genes is altered in these conditions, among which are several secreted proteins. The authors hypothesize that epidermal ribosome inhibition results in activation of a secreted signal and find evidence for the dense core vesicle regulator unc-31/CAPS being involved in the non-autonomous quiescence phenotype. Overall this work extends our understanding of ribosome function in vivo. The experiments are solidly performed and the data sets should be useful to others in the field. The main concerns are that the mechanism by which dense core vesicle signaling might be activated is not explored in depth. Other concerns are minor and could be addressed by toning down some claims or revising the presentation.

We thank the reviewer for their positive assessment of our study and for their constructive critique and valuable suggestions. We address all specific points below including the main concern related to the dense core vesicle signaling.

The authors find evidence dense core vesicle regulator unc-31 functions in the epidermis to mediate the quiescence response. This finding is slightly unexpected as most prior work on unc-31 expression (e.g. a recent study on unc-31 knockins from Pocock lab) indicated it was primarily expressed in the nervous system. The current study could be strongly improved by direct evidence (beyond public RNAseq data) that unc-31 is expressed in the epidermis, or whether epidermis has dense core vesicles by cell biological or ultrastructural criteria. The latter could be checked using public domain datasets. The study would also be strengthened by test of whether other DCV pathway genes (e.g. ida-1, etc) play similar roles to unc-31. It is noted that the observed effects of unc-31 inhibition are weak e.g. Fig 7E shows maybe a 5% change in body length. A slightly stronger effect is seen in unc-31's suppression of a different mutant, rps-23 (Fig 7A) but almost no details are provided about this experiment, so it is unclear if it bolsters the model that unc-31 is required for signaling from the epidermis. While promoters used for RNAi or AID transgenes are somewhat tissue specific, they are not absolutely so, cf. the single cell RNA data on col-10 in Fig 5D. Thus weak effects could potentially result from targeting of transcripts in other tissues. Overall the conclusions that unc-31 acts in the epidermis should be either toned down, or substantiated by further experiments.

We acknowledge the reviewer's concerns and have taken them into account in our revised manuscript. We have provided further clarification on the results (*page 14 lines 20-24*) and methods (*page 26 lines 24-29*) related to animals carrying *rps-23* loss of function mutation in the revised manuscript.

While endogenously tagging *unc-31* would provide direct evidence of its expression in the epidermis, these experiments are complicated by the large number of diverse isoforms of this gene (19 isoforms whose initiation sites span more than 5kb). Hence, we have decided to focus on providing complementary evidence regarding the involvement of the DCV pathway in the epidermis.

First, we generated strains with a single-copy reporter insertion driven by the promoter of *rab-3*, another component of the DCV, and an epidermal reporter driven by the *col-10* promoter. Our data revealed *rab-3* transcript expression in epidermal cells, supporting our contention of DCV pathway involvement in epidermal tissue (**New Fig S12A, S12B,** *page 16 lines 1-5*).

Second, as recommended, we have investigated the functional role of other dense core vesicle (DCV) pathway genes. Among the DCV pathway genes tested, we found that epidermis-specific knockdown of *ida-1* also suppressed the organism-wide growth quiescence induced by epidermal ribosome synthesis inhibition (**New Fig 8A**). We generated an endogenous fluorescent reporter knock-in strain for IDA-1, a DCV membrane protein with its C-terminus exposed externally on the vesicles, allowing us to visualize these DCV particles using confocal microscopy. We observed DCV puncta in or in close proximity to epidermal cells (**New Fig 8B)**. Although we cannot conclusively determine if these puncta are inside epidermal cells, we have carefully revised our results and discussion sections accordingly. We suggest that DCVs are present in or near epidermal cells (**New Fig 8B, New S12C**) and they are likely secreting growth inhibitory molecules in response to ribosome biogenesis-mediated stress, thereby controlling organism-wide growth (*page 16 lines 6-20; page 21 lines 8-15*).

Regarding the modest effects observed upon UNC-31 inhibition, we suggest that even these slight changes may have biological significance considering the multifaceted nature of growth regulation. We also agree that the tissue-specific promoters we used are not absolute in their specificity, thus, effects on other tissues could potentially contribute to the observed phenotype. Given these considerations, we rephrased our conclusions about UNC-31's action in the epidermis (*from page 20 line 29 to page 21 line 4*). We have therefore adjusted our language throughout the manuscript to reflect this uncertainty.

Presentation/wording

The authors show convincingly that the auxin-AID system is effective to deplete the worm versions of putative ribosome synthesis factors, but it is less clear if ribosome synthesis itself is inhibited (as stated in the title, and elsewhere) or if these genes have some other essential functions. There are two issues: first whether the worm orthologs have been investigated to assure that they act similarly to other systems, and second whether inhibition of these genes has the same consequences on ribosome populations. The authors observe overlap in effects on gene expression but without cell biological or biochemical analysis of ribosomes per se, it remains less clear what the effect of these gene inhibitions is in vivo. The nomenclature of these genes could also be clarified, e.g.

rrb-1 is not currently annotated as such in the worm database, but a similar gene rrbs-1 has been annotated (not mentioned in this work).

We thank the reviewer for bringing up these essential points. We have carried out further experiments to address the concerns and have updated our manuscript accordingly.

To substantiate our claim that the *C. elegans* orthologs of the ribosome synthesis factors indeed have an impact on ribosome synthesis, we have conducted polysome profiling experiments. First, when RPOA-2, a subunit of RNA pol-I, was depleted, we noticed a reduction in the peaks corresponding to all subunits as well as translating ribosomes, without any preferential decrease of a particular subunit (**Revised Fig S2C**). Second, when RRB-1, a large subunit biogenesis factor, was depleted, we noted reductions in the large subunit and monosomes, accompanied by an accumulation of the small subunit peak (**Revised Fig S2D**). Last, upon depletion of TSR-2, a small subunit biogenesis factor, we observed a significant decrease in mature ribosomes and a concomitant increase in the levels of the large subunit (**Revised Fig S2E**). These results (*from page 5 line 26 to page 6 line 7*) align with findings reported for their yeast orthologs (Iouk et al., *Molecular and Cellular Biology*, 2001; Schutz et al., *Elife*, 2014).

As for the nomenclature of *rrb-1* and *tsr-2*, we have contacted WormBase to request updates to the annotations. We believe that these additional experiments and clarifications provide further validation for the implications of our study.

The authors frame their findings as an example of tissue level coordination of growth analogous to the role of Dlip8 in Drosophila. However my understanding is that in the fly system inhibition of ribosome function slows developmental timing allowing growth over a longer period to reach a normal size, whereas in the C elegans work the result is quiescence / developmental arrest in all cases. It is not clear if the authors have demonstrated 'tuning' of ribosome synthesis that allows modulation of growth rate as opposed to arrest. These distinctions could be clarified in the discussion

We appreciate the reviewer's comment regarding the comparison to the Drosophila *Dilp8* study (Boulan et al., 2019, *PMID: 31006647*). In our investigation, we depleted ribosome biogenesis factors by employing the auxin system, which provides more robust reduction than RNAi, which was utilized in the *Drosophila* study. This fundamental difference in the methodology employed could explain the distinct outcomes observed, i.e., the developmental delay in Drosophila versus the developmental quiescence in *C. elegans.* We now explain these differences and the potential effects of varying experimental approaches in the discussion section of our manuscript to enhance understanding (*page 19 lines 12-18*).

The statistical analyses should be checked by a statistical expert. In some places (e.g. Fig 4A) it is unclear if there is any correction for multiple comparisons. The use of different y axes for each data plot is confusing and potentially misleading; bar charts should be consistent with a 'zero' baseline.

We have now consulted with a statistician to ensure the accuracy of our analyses. In response to the point about multiple comparisons (**Fig S3A, 4A-C, S7A, S7B, 8A, 8D**), we now include t-test results corrected for multiple comparisons (Bonferroni method).

Regarding the presentation of data, we have revised **Fig 4A**, and similar figures, to use a consistent y-axis scale and a 'zero' baseline for all bar charts. Nevertheless, to emphasize the narrow range where the change in body length occurs, we opted for a discontinuous y-axis. We marked the break on the chart clearly.

C. elegans jargon such as hypodermis could be replaced with 'epidermis' throughout, as it has long been accepted that nematode hypodermis is equivalent to epidermis. Moreover 'hypodermis' means something different in human skin biology.

Thank you for the suggestion. We agree with this comment and have accordingly revised the manuscript to replace 'hypodermis' with 'epidermis' throughout the text.

The work could be put in better context by citations of prior studies of possible signaling from the C. elegans hypodermis to other tissues e.g. Madaan et al 2020 on regulation of BMPs by collagens.

Thanks for the insightful suggestion. We have now included this citation in the discussion section of the manuscript (*page 18, lines 27-29*). The addition of this reference highlights the potential for signaling from the *C. elegans* epidermis to other tissues, which aligns with the findings of our study.
